# Survey of Lichenized Fungi DNA Barcodes on King George Island (Antarctica): An Aid to Species Discovery

**DOI:** 10.3390/jof9050552

**Published:** 2023-05-11

**Authors:** Renato Daniel La Torre, Daniel Ramos, Mayra Doris Mejía, Edgar Neyra, Edwin Loarte, Gisella Orjeda

**Affiliations:** 1 Laboratorio de Genómica y Bioinformática para la Biodiversidad, Facultad de Ciencias Biológicas, Universidad Nacional Mayor de San Marcos, German Amezaga 375, Lima 15081, Peru; 2 Dirección de Investigación en Glaciares, Instituto Nacional de Investigación en Glaciares y Ecosistemas de Montaña, Centenario 2656, Huaraz 02002, Peru; 3 Herbario Sur Peruano−Instituto Científico Michael Owen Dillon, Jorge Chavez 610, Arequipa 04001, Peru; 4 Facultad de Medicina, Universidad Peruana Cayetano Heredia, Honorio Delgado 430, Lima 15102, Peru; 5 Unidad de Investigación Genómica, Laboratorios de Investigación y Desarrollo, Universidad Peruana Cayetano Heredia, Honorio Delgado 430, Lima 15102, Peru; 6 Facultad de Ciencias del Ambiente, Universidad Nacional Santiago Antúnez de Mayolo, Centenario 200, Huaraz 02002, Peru

**Keywords:** Lichen-forming fungi, Admiralty Bay, DNA barcoding, diversity, *Austrolecia*, *Buellia*, *Lecidea*

## Abstract

DNA barcoding is a powerful method for the identification of lichenized fungi groups for which the diversity is already well-represented in nucleotide databases, and an accurate, robust taxonomy has been established. However, the effectiveness of DNA barcoding for identification is expected to be limited for understudied taxa or regions. One such region is Antarctica, where, despite the importance of lichens and lichenized fungi identification, their genetic diversity is far from characterized. The aim of this exploratory study was to survey the lichenized fungi diversity of King George Island using a fungal barcode marker as an initial identification tool. Samples were collected unrestricted to specific taxa in coastal areas near Admiralty Bay. Most samples were identified using the barcode marker and verified up to the species or genus level with a high degree of similarity. A posterior morphological evaluation focused on samples with novel barcodes allowed for the identification of unknown *Austrolecia*, *Buellia*, and *Lecidea* s.l. species. These results contribute to better represent the lichenized fungi diversity in understudied regions such as Antarctica by increasing the richness of the nucleotide databases. Furthermore, the approach used in this study is valuable for exploratory surveys in understudied regions to guide taxonomic efforts towards species recognition and discovery.

## 1. Introduction

Lichenized fungi are filamentous fungi (mycobionts) which associate with one or more alga and/or cyanobacteria (photobionts) to form stable symbiotic structures known as lichens. More than 19,000 ascomycete fungal species from several orders are obligate lichens with polyphyletic origins [1,2,3]. Due to the properties conferred by the symbiosis, lichenized fungi can be found in every major ecosystem, including those with extreme conditions such as Antarctica [4]. In these ecosystems, lichenized fungi play important roles as providers of numerous ecosystem functions and services [5]. For example, their thalli can harbor a rich microbial community and are thought to be a platform for the diversification of other fungal species [6,7].

Paramount to lichenized fungi research and diversity estimation is an accurate species delimitation. Recently, lichen species delimitation and identification shifted towards an integrative approach, i.e., using multiple and complementary sources of evidence [8,9]. As a taxonomy tool, molecular data can be used to evaluate current species hypotheses or propose putative species under a robust statistical framework, which can be further evaluated using additional evidence [10]. The inclusion of molecular data is valuable in scenarios where phenotypic characters are limited such as for cryptic species [9,11,12]; however, the establishment of more than 184 morphologically distinct species of the basidiolichen *Core* based on barcoding sequences exemplifies its potential for accurately assessing the biodiversity of even well-studied macrolichens [13,14].

When used as a complementary or main identification tool, DNA barcoding has been shown to be useful for some lichenized fungi species [15,16,17]. The conventional DNA barcode marker for Fungi is the internal transcribed spacer (ITS) [18]. Despite potential challenges arising for some groups when used as the only marker (e.g., intragenomic variation or lack of resolution for discrimination), the ITS marker remains a useful exploratory means to assess fungi species delimitation and identification [8,19]. For many species it may be necessary to include additional markers and interpret the phylogenetic results in an integrative context to obtain an accurate outline of their boundaries [20].

The success of DNA barcoding for species identification is directly related to the amount of information for a given group in a “taxonomic” setting and the area of study in a “floristic” setting [15]. As species identification using DNA barcoding relies on a stable taxonomy and reliable nucleotide repositories; its applicability is limited for understudied communities and habitats [8,21]. In that regard, Antarctic lichenized fungi diversity is far from fully characterized and early phenotypical documentations have been shown to be imprecise in light of new molecular evidence [22]. Furthermore, apart from limited taxon coverage, practical problems concerning sequence submissions to reference databases could hinder the achievement of a precise identification and call for a careful, individual analysis of sequence-based results [8,23,24].

High-throughput sequencing techniques have become increasingly common when characterizing both mycobiont and photobiont molecular diversity [25,26]. Compared to conventional Sanger sequencing, HTS allows for a far more reliable determination of the sequences within a mixed sample, making it useful for studying both the main lichen components but also the underlying thalli-associated microbiome [27,28,29,30]. DNA metabarcoding approaches have shown a high potential for lichen biodiversity assessments [31,32]; although an additional consideration to the ones mentioned above is to take caution when discriminating the target lichenized fungi from the “noise” generated by the presence of secondary fungi, biological variation and sequencing artifacts [29,31,33].

Previous studies involving DNA sequencing in Antarctica have been circumscribed to specific taxonomic groups, while others employed a “floristic” context. An early effort to identify lichenized fungi in a molecular survey context on King George Island was unsuccessful due to the limited completeness of repositories at the time [34]. Later on, molecular species delimitations conducted across Antarctica resulted in a better understanding of lichenized fungi diversity in this extreme environment, while at the same time contributing to increasing their sequence representativity in the databases [26,35,36,37].

In this study, we explored the lichenized fungi genetic diversity on King George Island using a floristic survey in combination with a DNA barcode obtained through Sanger sequencing. Our methodological approach resulted in the determination of a rich lichen diversity. The data gathered allowed us to assess whether the current information stored in the nucleotide databases allowed a direct, exact identification of lichenized fungi in an area without a previous phenotypic characterization. In addition, our “floristic” approach resulted in a description of species with previously unknown barcodes, which contribute to the enrichment of the current nucleotide databases and could lead future sampling efforts towards the discovery of taxonomic novelties.

## 2. Materials and Methods

### 2.1. Study Site and Handling of Samples

**Figure 1:**
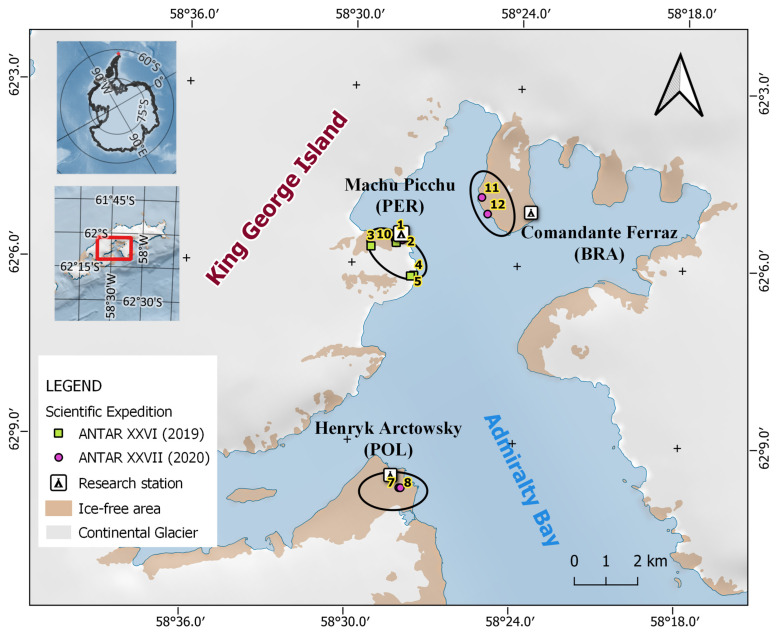
Sampling sites in this study. Lichens were collected in coastal areas near Admiralty Bay, King George Island, in three sites indicated by black ovals near research stations. Numbered green and pink points indicate transects used during scientific expeditions in 2019 and 2020, respectively. Information about each transect is detailed in Table 1.

Samples were collected on King George Island during the XXVI and XXVII Scientific Campaigns from Peru to Antarctica carried out in 2019 and 2020, respectively. Lichens were collected unrestricted to their taxon using transects in three sites along the coast of Admiralty Bay (Figure 1, Table 1). In 2019, six transects were used in the surroundings of Machu Picchu Scientific Base (Peru; transects 1–6). In 2020, two transects were used in each sampling site near the research stations Henryk Arctowski (Poland; transects 7 and 8), Machu Picchu (transects 9 and 10), and Comandante Ferraz (Brazil; transects 11 and 12). Small lichen fragments were collected, along with rocks or bryophytes cut using sterilized tools depending on how attached they were to the substrates. Rare or scarce lichens were not collected to avoid depleting the current lichen diversity.

All samples were placed in paper bags and stored in a dry thermic box before they were taken to the laboratory. There, the samples were sterilized using consecutive washes with ethanol 95%, sodium hypochlorite 0.5% and ethanol 70%, dried in a clean bench surface covered with paper towels and stored in individual sealed paper bags at room temperature until molecular processing.

### 2.2. DNA Extraction from Lichen Thalli

Up to 100 mg of lichen thalli from each sample was cut into pieces or scraped from its substrate into separate microtubes for DNA extraction with a modified protocol from Cubero and Crespo (2002) [38]. Both vegetative and reproductive material (when present) were used indistinctively, but small debris or substrate remains were removed. A small portion of 300 μm glass beads proportional to at least half the volume of the sample, and a single 5 mm steel bead were added to the microtube containing the tissue before exposing it to nitrogen liquid and shaking in a TissueLyser II for 1 min. This cycle of cooling and shaking was repeated two times before spinning down, adding a CTAB lysis buffer (100 mM Tris-HCl [pH 8.0], 30 mM EDTA, 1 M NaCl, 1% CTAB, 1% *w*/*v* PVPP) and incubating for 45 min at 70 °C, mixing by inversion every 10 min. For hard material such as thalli from *Usnea* species, adding more glass beads or an additional shaking cycle was needed before obtaining a fine powder. A purification step using chloroform−isoamyl alcohol (24:1) was performed and the upper phase was transferred to a new tube. Two volumes of CTAB precipitation buffer were added (40 mM NaCl, 0.5% *w*/*v* CTAB) and the supernatant discarded after centrifugation. Prewarmed 400 μL of 1.2 M NaCl was added to resuspend the pellet and then 2 μL of DNAse-free RNAse (10 mg/μL). After incubating for one hour at 37 °C, a new purification step using chloroform−isoamyl alcohol (24:1) was performed along with successive washes with isopropanol and ethanol as indicated in the original protocol [38]. The DNA was resuspended in 50–75 μL of TE buffer (0.1 mM Tris-HCl [pH 8.0], 0.1 mM EDTA) and stored at −20 °C for later use.

Genomic DNA concentration and quality was determined using a Nanodrop spectrophotometer and an agarose electrophoresis gel. The protocol was repeated with a higher amount of tissue when DNA yield or purity was low (depending on the sample availability). The DNA extraction was considered successful when at least 1 ng/μL was obtained along a minimum 260/230 absorbance ratio of 1.5. Results were reported as median and the first and third quartiles (Q1 and Q3, respectively).

### 2.3. PCR Amplification of Fungal Barcode Marker

The fungal ITS region (ITS1-5.8S-ITS2) was amplified using the fungi-specific primer combination ITS1F and ITS4 [39,40]. The master mix conditions were optimized for the GoTaq^®^ DNA Polymerase as follows: 1X GoTaq^®^ Reaction Buffer, 200 μM of each dNTP, 1.5 mM MgCl2, 0.7 μM of each primer, and 50 ng of template DNA. The cycling conditions in an Eppendorf thermocycler consisted in 1 cycle of 95 °C for 7 min; 30 cycles of 95 °C for 30 s, 56 °C for 1 min, and 72 °C for 30 s; and 1 cycle of 72 °C for 7 min. The amplicons were visualized in 2% agarose gels and purified with the Wizard^®^ SV Gel and PCR Clean-Up kit, either using the PCR product or a gel fragment containing a single band. The PCR was repeated with a higher amount of template DNA for samples showing a low-intensity band or negative initial amplification.

### 2.4. Sequencing and Sequence Analysis

A subset of amplicons was sequenced in both directions using Sanger sequencing in an external laboratory. The resulting sequences were manually edited using Sequencher 5.4.6 [41] or removed from the analysis depending on the quality of the electropherograms. A consensus was generated for samples with both forward and reverse sequences available. All sequences were submitted to GenBank under the accession numbers OP730747–OP830861.

Each sequence was independently subjected to a BLAST similarity search against GenBank using the *nblast* algorithm with default parameters except for the maximum number of alignments which was set to 500 [42]. Samples with sequences matching non-lichenized fungi were discarded from further analysis. BLAST matches were filtered using a minimum coverage and identity percentage of 80% and 95%, respectively; except for some sequences with no high-similarity association in which the minimum identity percentage used was 80%.

For the sequences identified as lichenized fungi in BLAST, a bootstrap consensus tree from a maximum likelihood analysis with 1000 bootstrap iterations was constructed in RaxML version 8 using an alignment with the ambiguous sites removed in GBlocks [43,44]. The GTR+I+G substitution model was selected for tree construction. Public sequences from the closest Species Hypothesis (SH) in the UNITE database [45] were included for unique samples in this dataset. As the purpose of this analysis was to cluster the samples rather than to infer phylogenetic relationships, no outgroup was included.

### 2.5. Sequence-Based Grouping and Identification

The samples were grouped based on their filtered BLAST hits and maximum likelihood analysis results. An initial identification up to the species or genus was made only for groups with BLAST hits with high identity percentages available based on the top match or lowest common ancestor (LCA). These identifications were verified or, in few cases, improved a posteriori through the search of morphological diagnostic characters. On the other hand, for groups with no high-similarity BLAST associations (i.e., samples with novel barcodes), a morphological description of the thallus and reproductive structures was carried out in order to establish their taxonomic classification.

## 3. Results and Discussion

### 3.1. SAMPLE Heterogeneity and Molecular Processing Performance

A total of 305 lichen samples displaying a varied morphological diversity were collected on King George Island during two scientific expeditions (Figure 1, Table 1). As the sampling was restricted to three sites in coastal areas in Admiralty Bay because of the difficulty to access inland areas, and perceived scarce lichens were not collected, the lichens in this study may not represent the full diversity on King George Island. Most of the samples were recognized as small crustose lichens difficult to identify without an in-field taxonomist. Some common lichen types such as *Usnea antarctica*/*U. aurantiacoatra* and *Placopsis antarctica* were readily recognized because of their distinctive macromorphological characteristics. Crustose or fruticose lichens with grey to white granular and squamulose scattered thalli were recognized as either *Stereocaulon* or *Ochrolechia* species, frequently found on top of bryophytes and mixed with other lichens. Few foliose lichens recognized as *Umbilicaria* or *Xanthoria elegans* were found.

**Figure 2:**
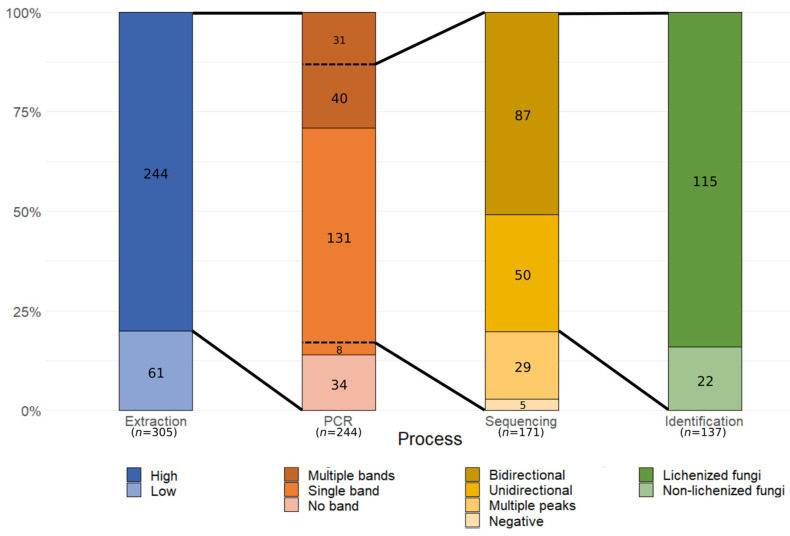
Molecular processing performance for each process applied to lichens collected in a floristic setting. Extracted DNA not meeting the criteria indicated in the Materials and Methods section were classified as “Low”, while the others as “High”. PCR results were classified according to their band pattern in an electrophoresis gel; only a subgroup of positive bands was used for sequencing. Sequencing electropherograms were classified as “Negative” when no peak was observed, “Multiple peaks” when both forward and reverse sequences showed more than two peaks for most sites, and “Unidirectional” and “Bidirectional” based on the observation of clear, high-quality peaks for one or both directions, respectively. Identification results are detailed in the text. The total number of samples for each process is indicated at the bottom of each bar in parenthesis.

The DNA extraction protocol used in this study showed a high degree of universality, as it resulted in DNA with yields (median: 30.43 ng/µL, Q1: 9.45 ng/µL, Q3: 91.11 ng/µL) and purity levels (median: 1.83, Q1: 1.75, Q3: 1.91) appropriate for successive assays for 80% (244/305) of samples independently of their morphology and/or type (Figure 2). Most of the samples from which DNA could not be extracted or which did not meet the minimum criteria were small fragments with minimal input thalli available (<6.5 mg). Using a more sensible method or commercial kits may be more effective for scarce samples with a minimal amount of material for DNA extraction.

PCR amplification using a fungi-specific primer combination resulted positive for 86% (210/244) of genomic DNA samples (Figure 2). There was no apparent correlation between either lichen characteristics or input DNA quantity or purity with PCR amplification success; however, preliminary assays using an alternative enzyme with higher specificity and sensitivity yielded improved results, suggesting the carryover of inhibitors after DNA extraction for those samples with no amplification. From the PCR-positive group, 139 samples displayed a single band in an electrophoresis assay, while the remaining showed two or more bands (Figure 2). A gel separation method was used for samples with multiple bands, although some were too close to each other to be effectively separated. The source of these co-amplifications were secondary fungi, either contaminants or part of their associated microbial community.

Amplicons from 171 samples were selected for purification and sequencing by Sanger, resulting in the barcode marker successfully sequencing in both directions for 87 samples, and only either forward or reverse direction for 50 samples (Figure 2). The rest of the samples showed an intermediate to high degree of noise.

Obtaining high-quality sequences from mixed samples using Sanger sequencing can prove challenging because of the presence of secondary targets. Previous studies addressing the use of this technique for sequencing lichenized fungi reported similar difficulties on many of the taxa evaluated [25,46,47]. An explanation for the low sequencing success is the variable presence of secondary fungi conforming a mycobiome [29], but also other lichens in the form of small fragments or spores. To effectively obtain the target mycobiont sequence, multiple studies have used HTS techniques such as 454-pyrosequencing; however, due to the high rate of sequencing errors, Sanger references are recommended [33,48]. Illumina sequencing has also been used to obtain the barcode marker of lichenized fungi with a better success in comparison to Sanger sequencing. In a previous study comparing both techniques for historical and fresh basidiolichen *Cora* samples, only 58% of fresh samples could be sequenced using Sanger sequencing, although the authors discuss their typical overall success being 70–90% in previous attempts at sequencing fresh material [30]. These results are similar to the 67.3% (115/171 samples) success when sequencing lichenized fungi using conventional Sanger sequencing in this study (Figure 2); the identification results are detailed in the next section.

It is important to note that Sanger sequencing success has been shown to be associated with the relative abundance of secondary targets [25]; however, this can vary greatly between taxa [25,29]. In this study, no association could be found between sequencing outcome and recognized taxa.

Even for non-mixed samples, sequencing can be challenged by the presence of biological sequence variation. The ITS marker is a subregion of the rDNA cistron, which is repeated multiple times across the fungal nuclear genome. This can lead to intragenomic variation, which has been reported for some lichens due to incomplete lineage sorting [17,33]. Furthermore, intra-sample rather than intragenomic variation is a possibility when studying multicellular organisms. Independently of the source of variation, this could lead to difficulties in species identification through DNA barcoding, particularly on rapidly evolved species [49]. Since Sanger sequencing alone is limited for detecting intragenomic sequence variation, and we included samples from a broad taxonomic range rather than closely related species, we deemed it unlikely that these variations have a significant effect on the initial molecular identification results.

Although in this study a gel separation technique was used for the separation of multiple bands, some single bands still yielded low-quality sequencing electropherograms, probably due to the presence of additional targets with similar or identical length. Additional techniques such as molecular cloning are effective for isolating and sequencing amplicons in mixed samples. Alternatively, target mycobionts could be isolated through microbiological culture methods prior to DNA extraction and sequencing (e.g., [50]). While these techniques may improve the number of sequences suitable for identification purposes in exploratory studies, they may be costly and difficult to apply in laboratories lacking the right equipment or infrastructure.

Another possible approach to working with mixed samples is to increase the amplification specificity by designing taxon-specific primers; however, this was impractical in this study as the sampling was unrestricted to specific taxa and some specimens were not easily recognized with a macroscopical inspection.

Overall, due to the extent of the sampling and the universality of the DNA extraction, a large number of barcodes were recovered. As the amount of material needed for molecular processing is small, most of the samples were suitable for posterior morphological verification.

### 3.2. Lichenized Fungi Identification

From the 137 samples sequenced in this survey, 22 were associated in BLAST with non-lichenized fungi entries *Aspergillus* (11), *Athelia bombacina* (1), *Geltingia associata* (1), unnamed uncultured fungi (8), and an unknown basidiomycete (1) with varying degrees of similarity. Some of these species could be endo- or epilichenic fungi living naturally in the lichen thalli, natural contaminants that persisted after the sterilization at the laboratory, or contaminants originated during the manipulation of samples. For example, species from the cosmopolitan cold-adapted fungal genus *Aspergillus* are abundant in the Antarctic atmosphere and soil [51]. As discussed before, non-lichenized fungi interfered with the sequencing as additional targets co-amplified in the PCR; however, in some cases secondary lichenized fungi were also found as secondary products.

**Figure 3:**
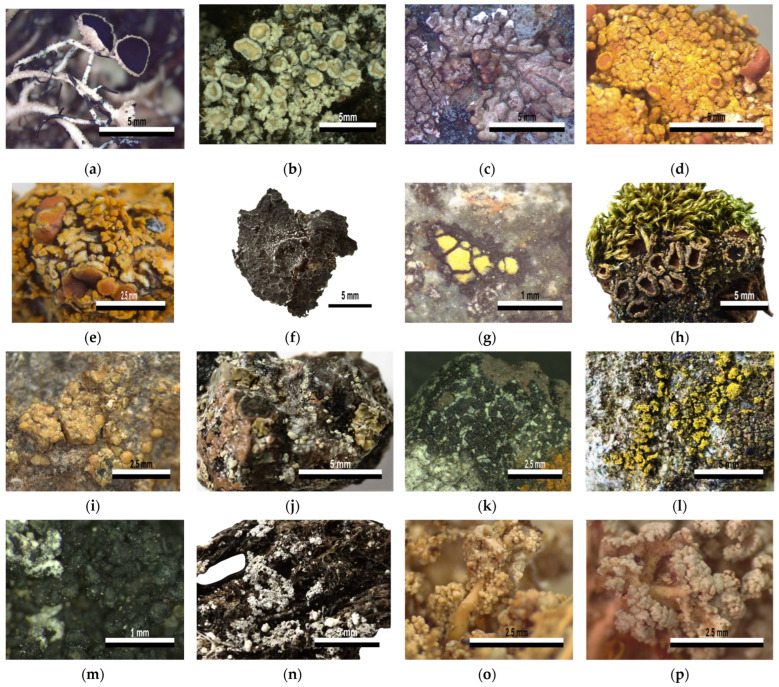
Lichenized fungi diversity on King George Island identified with an initial DNA-based approach. All samples were grouped based on their DNA barcodes. The respective families are indicated in parenthesis. (**a**) I: *Usnea aurantiacoatra* (Parmeliaceae), (**b**) II: *Ochrolechia frigida* (Stereocaulaceae), (**c**) III: *Placopsis antarctica* (Trapeliaceae), (**d**) IV-a: *Polycauliona regalis* (Teloschistaceae), (**e**) IV-b: *Caloplaca* sp. (Teloschistaceae), (**f**) V-a: *Umbilicaria decussata* (Umbilicariaceae), (**g**) VI-a: *Rhizocarpon* aff. *geographicum* (Rhizocarpaceae), (**h**) VII-a: *Psoroma hypnorum* (Pannariaceae), (**i**) VIII-a: *Lecanora polytropa* (Lecanoraceae), (**j**) VIII-b: *Lecanora* sp. s.l. (Lecanoraceae), (**k**) IX: *Lecidella carpathica* (Lecanoraceae), (**l**) X: *Candelariella flava* (Candelariaceae), (**m**) XI: *Steinera intricata* (Koerberiaceae), (**n**) XII: *Lepraria* sp. (Stereocaulaceae), (**o**) XIII-a: *Stereocaulon* sp. 1 (Stereocaulaceae), (**p**) XIII-b: *Stereocaulon* sp. 2 (Stereocaulaceae).

The remaining 115 samples were identified as lichenized fungi and were assigned to 23 groups according to the BLAST similarity search and maximum likelihood analysis results; samples identified as sharing a closer taxonomic level were clustered together (Figure 3, Table 2).

The groups with significant BLAST matches included samples spanning 15 families, in 10 orders of the class Lecanoromycetes and one of Candelariomycetes (Figure 3; Table 2 and Table S1). As detailed above, these samples were initially identified based on their BLAST top hits, and the identification was then verified by looking for characteristic morphology features of the genus or species. A large proportion of samples commonly found were identified as *Usnea antarctica*/*U. aurantiacoatra* (27 and 11, respectively) and *Placopsis antarctica* (24). Samples of common fruticose muscicolous fragments with similar grey to white granular or squamulose scattered thalli, although frequently not showing reproductive structures, were identified as either *Ochrolechia frigida* (13) or *Stereocaulon* sp. (4). Foliose samples were identified as *Umbilicaria decussata* (2) or *Umbilicaria* sp. (2). Although the small crustose samples were difficult to recognize, the barcodes aided in the identification of *Steinera intricata* (7), *Rhizocarpon* aff. *geographicum* (3), *Psoroma hypnorum* (2), *Candelariella flava* (2), *Lecidella carpathica* (2), *Lecanora* sp. sensu lato (2), *L. polytropa* (1), *Caloplaca* sp. (1), *Polycauliona regalis* (1), *Lepraria* sp. (1), and *Myriospora* sp. (1).

On the other hand, nine samples had no high-similarity BLAST associations as their barcodes were new to GenBank (Table 3 and Table S1). A morphology evaluation of these samples allowed the identification of *Austrolecia* sp. (4), *Lecidea* sp. sensu lato (2), *Buellia* sp. sensu lato (1), *Rhizocarpon* sp. (1), and *Psoroma hypnorum* (1).

#### 3.2.1. DNA-Based Identification at the Species Level

An initial BLAST analysis with a posterior morphological confirmation allowed the identification of eleven sample groups unambiguously at the species level (I, II, III, IV-a, V-a, VI-a, VII, VIII-a, IX, X, XI; Table 2). All samples in group I were associated with both *Usnea antarctica* and *U. aurantiacoatra* (38 samples). This reflects the lack of resolution of the fungal barcode to distinguish between these known species pairs (i.e., morphs of lichens which only differ phenotypically by their reproductive strategies [10]). In a previous study, sequence-based data alone were insufficient to separate these species in two lineages, and the use of microsatellite data was necessary to evidence a clear genetic distinction [52]. An examination of the presence or absence of apothecia allowed a species-level identification for specimens in this group (Figure 3a).

Similarly, all samples included in group II had sequences associated with both *Ochrolechia frigida* and *O. tartarea* after the BLAST analysis (Table 2). Examining these BLAST associations, the two entries in GenBank labeled as *O. tartarea* were obtained from samples collected in Antarctica and China, and are highly similar to the other *O. frigida* entries. However, previous phylogenetic studies on Ochrolechiaceae clearly separated both species using either mitochondrial SSU and nuclear LSU markers, or ITS sequences [53,54,55]. It is not possible to determine whether the two *O. frigida* entries associated with the samples in this study were mislabeled or are outdated; however, it is reported elsewhere that *O. frigida* material showing no spines is often labeled as *O. tartarea* [56]. A main difference between both taxa is in their chemistry; however, given the previously reported clear genetic difference between both taxa, and the fact that almost all specimens showed the characteristic spines of *O. frigida* (Figure 3b), we identified these samples as such.

Samples in group III readily recognized as *Placopsis antarctica* were associated with entries of *P. antarctica* and *P. parellina* collected in Antarctica (Table 2). The only ITS entry in GenBank labeled as *P. parellina* (AY212822) is most likely outdated as it was submitted in 2004, one year before *P. antarctica* was described [57,58]. In the secondary curated database UNITE, this particular entry is yet to be renamed, although it is placed in a *P. antarctica* SH at a 0.5% threshold. All 24 samples in this group were confirmed to be *P. antarctica* because of their characteristic central cephalodia, whitish thallus and presence of sorediate pits (Figure 3c). Likewise, the only sample in group IV-a only matched entries labeled as *Caloplaca regalis* and *Gondwania regalis* (Table 2), which are currently synonyms of the valid name *Polycauliona regalis* according to the Species Fungorum database (2022). This and other species of the genus have been reported in Antarctica near bird colonies with a fruticose habit and a usual orange thallus (Figure 3d), which reacts with K+ becoming purple–red [59].

The group V-a included samples associated with *Umbilicaria decussata* and *U. krascheninnikovii* (Table 2). A recent revision of the phylogenetic relationships within Umbilicariaceae using multiple markers reported the inclusion of *U. krascheninnikovii* on the *U. aprina* group, separated from the *U. decussata* group [60]. However, the *U. krascheninnikovii* entry in particular, which was associated with the barcodes in group V-a (AY603134.1), has consistently been found to be related to *U. decussata* in previous works [61,62,63] and was subsequently revised as *U. decussata* in UNITE (SH1883021.08FU). The possibility of mislabeling as well as the presence of distinctive peripheral white ridges on the upper surface of the samples indicated that these corresponded to *U. decussata* (Figure 3f).

The group VI-a included three samples with sequences associated in BLAST with two species in the *Rhizocarpon geographicum* complex, *R. geographicum* and *R. nidificum* (Table 2), the latter being endemic to Antarctica [64]. This complex is included along with other three groups of yellow species in the subgenus *Rhizocarpon* and has been proposed as a single highly plastic species on the basis of molecular and phenotypic revisions; however, further in-depth studies are needed to validate this idea [65,66]. The scarce material collected in this study allowed the identification of the crustose yellow areoles embedded in a black prothallus characteristic of these species (Figure 3g). 

The remaining identifications at the species-level include two samples in the group VII-a exclusively associated in BLAST, and identified as *Psoroma hypnorum* (Figure 3h; Table 2), as opposed to a sample with a closely related sequence in group VII-b which matched with different *Psoroma* species with a low degree of similarity (Table S1). Additionally, one sample in group VIII-a had a sequence associated with a *Lecanora polytropa* entry (Figure 3i; Table 2); two Samples in group IX matched *Lecidella carpathica* (Figure 3k; Table 2); two samples in group X were associated in BLAST and identified as *Candelariella flava* (Figure 3l; Table 2); and seven samples in group XI were identified as *Steinera intricata* with no other significant BLAST association (Figure 3m; Table 2). For this last sample group, some of the sequences were obtained from samples recognized as *Ochrolechia frigida* or *Psoroma hypnorum*, raising the possibility that spores or small fragments were co-amplified as these lichens are often found intertwined along other lichens and bryophytes.

#### 3.2.2. DNA-Based Identification at the Genus Level 

Seven sample groups were identified up to the genus, being ambiguous at the species level (IV-b, V-b, VIII-b, XII-a, XII-b, XIII, XIV; Table 2). The ambiguous identification of groups IV-b and XII containing one sample each, to *Caloplaca* sp. and *Lepraria* sp., respectively, can be attributed to a lack of information in databases, as both of these groups matched to different species with identity percentages up to 97.5% (Table 2). General morphological features such as thallus color and form confirmed the genus identification of both groups: orange lobes, mostly applanate, for *Caloplaca* sp., and a leprose granular thallus without displaying any reproductive structure for *Lepraria* sp. (Figure 3e,n).

The sample group V-b identified as *Umbilicaria* included two samples with sequences matching entries labeled *U. aprina* and *U. africana* (Table 2). Both of these species were reported to be closely related within the *U. aprina* group in a phylogenetic revision of the relationships within Umbilicariaceae [60]. This genus was readily verified due to the samples displaying the characteristic brown foliose thallus attached by an umbilicus (Figure 3f). Similarly, the sample group VIII-b identified as *Lecanora* sp. sensu lato included two samples whose sequences had significant BLAST associations with *Rhizoplaca aspidophora*, *Lecanora fuscobrunnea* and *L. polytropa*. The phylogenetic relationship of *Rhizoplaca* being nested within the highly polyphyletic *Lecanora* sensu lato group is widely documented both using morphological and genetic information [67,68]. Given these associations with various species within the complex, and the observation of a pale-yellow crustose thallus of the single sample in this group, it was identified as *Lecanora* sp. s.l. (Figure 3j).

Four samples in group XIII had sequences matching entries of various fruticose species from the genus *Stereocaulon* subgenus *Stereocaulon* section *Stereocaulon* [69]. These matches included entries labeled as *S. alpinum*, *S. grande* and *S. saxatile* for the two samples in group XIII-a, and *S. glabrum* and numerous unidentified *Stereocaulon* species for the two samples in group XIII-b. The unresolved relationship between these and other species in the *Stereocaulon* clade was shown in previous studies examining their phylogenetic relationships, in which additional nuclear markers were also included [69,70,71]. Despite the barcode marker not being resolutive enough to attain an exact identification, a morphological evaluation allowed us to not only verify the genus *Stereocaulon*, but also to differentiate two different species corresponding with their DNA-based grouping. The sample group XIII-a had yellowish spheroid phyllocladia (Figure 3o), while the sample group XIII-b displayed white flattened phyllocladia (Figure 3p).

For all the groups mentioned above, the species conceptualized within each taxonomic group may not be correlated with clearly separated lineages. Therefore, it is expected that the barcode marker alone would not be resolutive enough to attain a species-level identification. Although we recognize that including the evaluation of additional phenotypic markers such as chemistry would be valuable to attain a more exact identification for these groups, our results still illustrate how an initial DNA-based approach can narrow the list of possible options for identification and could serve as a starting point to guide the search for specific diagnostic characters.

Lastly, the ambiguous identification of the only sample in group XIV to *Myriospora* was due to its association both with *M. signyensis* and unidentified Lecanoromycetes entries with high identity percentages (Table 2). This sample consisted of scattered and small black apothecia on a rock substrate without thallus. Given that all of this scarce material had to be collected to have a significant amount for DNA extraction (still resulting in only 3.4 mg of tissue), a further morphological verification was not possible.

#### 3.2.3. Unidentified Lichenized Fungi Groups

The remaining five groups could not be identified using a DNA-based approach since none of the sequences had significant BLAST matches (Table 3 and Table S1). The samples in groups VI-b and VII-b matched multiple species from the genus *Rhizocarpon* and *Psoroma*, respectively. Despite the low identity percentages for these groups (below 88.9% for group VI-b, and 94.5% for group VII-b), their exclusive association in BLAST with species from the same genus in each case indicated that they may be under that classification. A morphological evaluation was performed for both of these groups. In the case of group VII-b, the morphological features similar to those of the two samples in group VII-a allowed the identification of *Psoroma hypnorum* (Figure 3h; Table 3)

**Figure 4:**
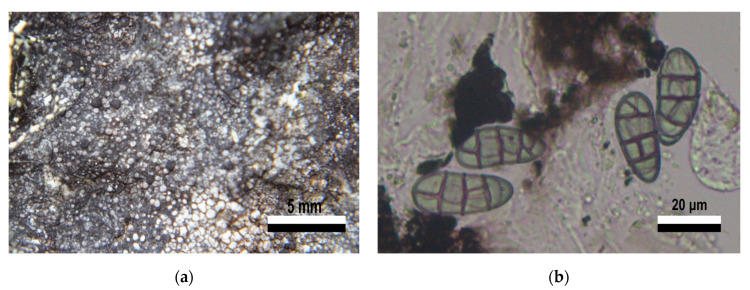
Morphology features from specimen (51A6) in group VI-b. *Rhizocarpon* sp., Rhizocarpales. (**a**) Crustose thallus, scattered areoles, sessile lecideine apothecia. (**b**) Submuriform gray ascospores.

The sample in group VI-b identified as *Rhizocarpon* sp. displayed a gray or light green thallus on a notorious black prothallus, areoles scattered on the substrate, and lecideine and sessile apothecia (Figure 4a). The gray, submuriform ascospores were 28–32 µm long, with up to four transverse and one longitudinal septa (Figure 4b). *R. nidificum* has most of these characteristics, but the color of the thallus is a more intense green–yellow, and the ascospores between 15 to 30 µm long. Another species with similar characteristics is *R. grande*, which has a gray thallus, and a black, inconspicuous prothallus. It is described as having submuriform ascospores, with lengths greater than 32 µm. In both cases, a comparison of characteristics to confirm the identity of this sample was not possible, as no pictures of the type material were found. A more detailed analysis and comparison with the type material of both species is required for a more exact identification.

The three remaining groups (XV, XVI, XVII) had sequences matching entries in GenBank with identity percentages below 90% and could only be recognized with a deep taxonomic level given the variety of matches (Table 3 and Table S1). These associations are not meaningful for a recognition based solely on molecular information and, at most, indicate that the barcodes are from lichenized fungi. A morphological evaluation of these specimens was needed to attain a more exact identification and elucidate whether they are previously recorded species for which there is no molecular information, or if they are undescribed taxa.

**Figure 5:**
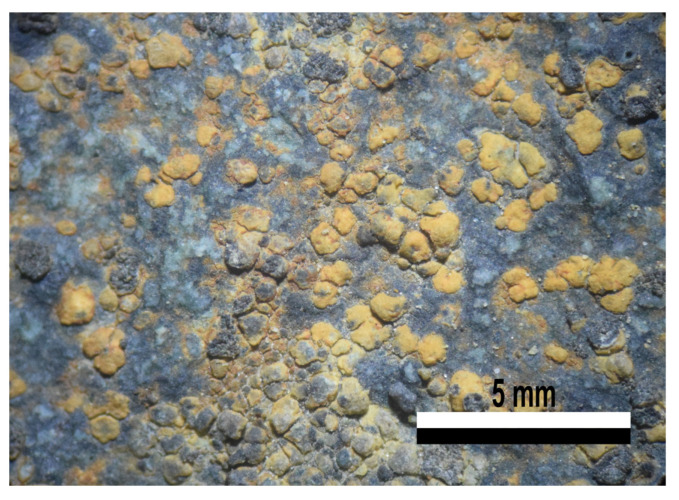
Specimen (111A) in group XV. Lecideales (*Lecidea* s.l.). Sterile crustose thallus.

The two samples in group XV were identified as *Lecidea* sp. s.l. These samples are characterized by an areolate, reddish-brown thallus. No reproductive structures such as apothecia, soredia or isidia were observed (Figure 5). These characteristics, as mentioned by Øvstedal and Lewis Smith (2001) [64], suggest that these specimens are *Lecidea silacea*; a taxon that was originally described in the Scandinavian region of northern Europe. It is possible that the individuals described in Antarctica were assumed to be the same taxon based on the similarity in color and overall shape of the thallus, therefore having a bipolar distribution similar to other taxa. Although the samples in this study did not result in high-similarity BLAST associations with any of the *L. silacea* entries already in GenBank (Table S1), not one of those entries correspond to samples collected in Antarctica. However, given that a misidentification could be more harmful for future identifications than an inexact labeling, it was decided based on this morphological evaluation to identify these two samples as *Lecidea* sp. s.l.

**Figure 6:**
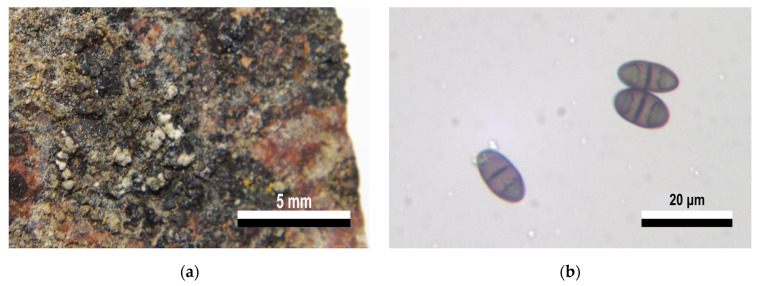
Morphology features from specimen (24C) in group XVI. Caliciales (*Buellia* sp. s.l.). (**a**) Crustose thallus, scattered areoles, lecideine apothecia at the same level as the thallus (immersed). (**b**) Brown polarilocular “adult” ascospores (40×).

The only sample in group XVI was identified as *Buellia* sp. s.l. This sample is characterized by having a crustose thallus with thick areoles and a cream to slightly green color, with some darkened areas (Figure 6a). Its lecideine apothecia are flat with slightly conspicuous edges. An important characteristic is the presence of “adult” polarilocular ascospores, that is, with a thickened central septum, ca. 8–10 µm (Figure 6b). Both Redón (1985) [72] and Øvstedal and Lewis Smith (2001) [64], mention that two *Buellia* species have this characteristic, namely *B. melanostoma* and *B. perlata*; however, this is only found in “juvenile” ascospores. Later in the development, they take on the typical characteristics of the genus, having thin ascospore septa and walls. *Amandinea insperata*, present in mountainous areas of the tropics and subtropical regions [73], also have polarilocular ascospores similar in size (16–18 µm × 6–9 µm) to those observed in the analyzed material. Additionally, *Amandinea* is characterized by its elongated, bacillary conidia, while *Buellia* has shorter, ellipsoidal conidia; however, these structures were not observed in the available material. The genus *Orcularia* was also considered, but a notorious thalline exciple of *Orcularia*-type ascospores [74] was absent, and the lack of lumens resembling small “bones” indicates a different ascospore ontogeny. Based on the presence of polarilocular adult ascospores, this sample would correspond to an undescribed taxon in the genus *Buellia* s.l. A future study including more abundant and larger samples from King George Island is pending to obtain a complete and detailed description of the total phenotypic characteristics to formally propose a new taxon.

**Figure 7:**
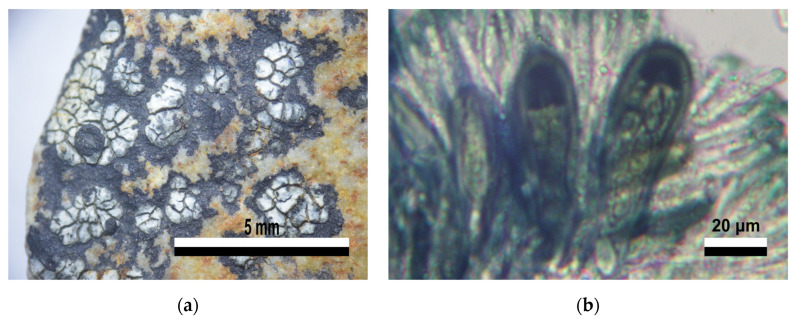
Morphology features from specimen (94A1) in group XVII. *Austrolecia* sp. Lecanorales. (**a**) Crustose thallus with scattered areoles surrounded by a black prothallus, with sessile lecideine apothecia. (**b**) *Catillaria*-type asci, simple and hyaline ascospores.

Finally, the four samples from the group XVII were identified as an unknown *Austrolecia* species. These samples are characterized by their crustose thallus dispersed on the substrate or forming small groups and with a noticeable black prothallus; lecideine, convex apothecia with a very thin edge, inconspicuous or even absent in some segments (Figure 7a). The hymenium is hyaline, with a blue–greenish epihymenium, (8) simple and hyaline ascospores, *Catillaria*-type asci and hyaline subhymenium (Figure 7b). Taken together, these characteristics indicate that the samples belong to the *Austrolecia* genus [64], under which only one species, *A. antarctica*, is currently described [75]. *A. antarctica*, as mentioned by Øvstedal y Lewis, presents several characteristics described for the samples in group X in this study; most noticeable, the thin or absent edge in mature apothecia, and ascospores being simple or showing a septum. However, the description also indicates a light brown hypothecium, which was found to be hyaline for the analyzed samples. The type material for *A. antarctica*, available in the digital database of the Herbarium of University of Helsinki, through JSTOR Global Plants, also shows that *A. antarctica* has a thallus with areoles more closely grouped, a less developed prothallus and more apothecia. Thus, it is possible that the analyzed material may correspond to an undescribed taxon in *Austrolecia*. The lack of a high-similarity BLAST association of these samples to any of the *Austrolecia* sequences from different areas in Antarctica already available in GenBank (Table S1) highlight the need for a thorough collection in future studies focused on this group.

### 3.3. Value of Molecular Surveys in Understudied Regions

The lichenized fungi survey using DNA barcodes presented in this study resulted in a variety of sequences from King George Island, nine of them completely new to GenBank (Table 3). The approach used did not rely on an initial phenotypic identification, thus allowing the posterior taxonomic efforts to be focused on the description of specimens filling a gap on the current nucleotide databases, or potential new taxa. In that regard, this strategy is to be considered for biological inventories unrestricted to a particular taxonomic group, particularly in understudied regions where the potential for species discovery is high.

The analysis of sequences from a community in understudied regions rely on a complete regional nucleotide database. In that regard, we are far from having complete DNA information in remote areas; however, the application of a DNA-based survey can lead to further sampling expeditions toward taxonomic novelties.

While Antarctica is a relatively understudied continent because of the limitations to exploration imposed by the harsh conditions, countries in the southern hemisphere harboring a vast proportion of the world’s biodiversity are in an equal or even greater need for biodiversity explorations. For these countries, the political priorities for research may not be in line with a thorough characterization of genetic diversity when there is no apparent conservation or immediate application objective. Additionally, local research groups may be discouraged from initiating such explorations and collections when there is little incentive for exploration of biodiversity, and a lack of infrastructure and personnel with taxonomic expertise [76].

Molecular explorations can function as a complementary tool to aid taxonomic work in light of a taxonomic impediment, because collecting a potential new species is a step further in reducing the taxonomic gap [77]. For example, having complete information about an underexplored group could allow an initial delimitation of potential species based on molecular phylogenies, which would be later tested with phenotypic features [9]. For this effort to be useful, rather than detrimental, to the current taxonomic challenges, it is important that the specimens are properly submitted to public repositories for future assessments.

Overall, these findings highlight the utility of an exploratory survey of lichenized fungi without a prior morphological assessment, and contribute to increasing the known genetic diversity on King George Island. Further detailed taxonomic revisions including the potential new species from King George Island presented here will aid in future taxonomic studies involving Antarctica’s lichenized fungi diversity.

## Figures and Tables

**Table 1:** Study site information. Sample size and coordinates are indicated for each transect.

Site Name	Transect Number	Sample Size	Coordinates		
			Latitude	Longitude	Elevation (m)
Scientific Campaign ANTAR XXVI–2019					
Machu Picchu Base	1	3	62°5′32″ S	58°28′9″ W	1
	2	9	62°5′32″ S	58°28′15″ W	2
	3	32	62°5′43″ S	58°29′19″ W	201
	4	6	62°6′12″ S	58°27′46″ W	25
	5	48	62°5′38″ S	58°28′25″ W	41
	6	24	62°5′40″ S	58°28′26″ W	44
Scientific Campaign ANTAR XXVII–2020					
Henryk Arctowski Base	7	27	62°9′48″ S	58°28′5″ W	46
	8	30	62°9′48″ S	58°28′2″ W	41
Machu Picchu Base	9	25	62°5′36″ S	58°28′12″ W	21
	10	30	62°5′36″ S	58°28′28″ W	38
Comandante Ferraz Base	11	46	62°4′51″ S	58°25′20″ W	48
	12	25	62°5′7″ S	58°25′7″ W	53

**Table 2:** Identification of samples (*n* = 106) based on a BLAST similarity search and a posterior morphology confirmation. Minimum BLAST coverage and identity percentages were 80% and 95%, respectively. Full list of matches available in Table S1.

Sample Group	BLAST Top Hits	Identity Percentage Range	Number of Samples	Identification
I	*Usnea aurantiacoatra*	98.36%−100%	11	*Usnea aurantiacoatra*
	*Usnea antarctica*	98.38%–100%	27	*Usnea antarctica*
II	*Ochrolechia frigida*	95.12%–100%	13	*Ochrolechia frigida*
	*Ochrolechia tartarea*	95.12%–100%		
III	*Placopsis antarctica*	98.2%–100%	24	*Placopsis antarctica*
	*Placopsis parellina*	99.44%–100%		
IV-a	*Caloplaca regalis*	99.62%	1	*Polycauliona regalis*
	*Gondwania regalis*	99.03%–99.4%		
IV-b	*Gondwania* sp.	97.23%	1	*Caloplaca* sp.
	*Caloplaca sublobulata*	96.25%		
	*Gondwania sejongensis*	96.01%		
V-a	*Umbilicaria krascheninnikovii*	99.79%–100%	2	*Umbilicaria decussata*
	*Umbilicaria decussata*	95.59%–98.79%		
V-b	*Umbilicaria aprina*	95.21%–100%	2	*Umbilicaria* sp.
	*Umbilicaria africana*	95.56%–99.8%		
VI-a	*Rhizocarpon geographicum*	95.02%–100%	3	*Rhizocarpon* aff. *geographicum*
	*Rhizocarpon nidificum*	99.75%–100%		
VII-a	*Psoroma hypnorum*	95.88%–100%	2	*Psoroma hypnorum*
VIII-a	*Lecanora polytropa*	99.67%	1	*Lecanora polytropa*
VIII-b	*Lecanora polytropa*	95%–98.32%	2	*Lecanora* sp. s.l.
	*Lecanora* cf. *polytropa*	95.41%–98.71%		
	*Rhizoplaca aspidophora*	100%		
	*Lecanora fuscobrunnea*	97.35%–99.1%		
IX	*Lecidella carpathica*	97.34%–99.73%	2	*Lecidella carpathica*
X	*Candelariella flava*	96.79%–99.45%	2	*Candelariella flava*
XI	*Steinera intricata*	95.61%–100%	7	*Steinera intricata*
XII	*Lepraria elobata*	95.71%–97.46%	1	*Lepraria* sp.
	*Lepraria caesioalba*	95.77%–97.45%		
	*Lepraria granulata*	97.35%		
	*Lepraria neglecta*	95.09%–97.34%		
XIII-a	*Stereocaulon alpinum*	95.11%–100%	2	*Stereocaulon* sp. 1
	*Stereocaulon grande*	99.32%		
	*Stereocaulon saxatile*	96.10%–99.13%		
XIII-b	*Stereocaulon glabrum*	97.08%–100%	2	*Stereocaulon* sp. 2
	*Stereocaulon* sp.	95.01%–100%		
XIV	*Myriospora signyensis*	95.33%–99.8%	1	*Myriospora* sp.
	Lecanoromycetes sp.	99.37%–99.55%		

**Table 3:** Samples (*n* = 9) with novel DNA barcodes discovered in this study. These samples had no significant BLAST associations, so the identification was made based on a morphological evaluation of thalli and reproductive structures. Full list of non-significant BLAST matches in Table S1.

Sample Group	Number of Samples	Sample Codes	Morphological Determination	GenBank Accession Codes
VI-b	1	51A6	*Rhizocarpon* sp.	OP730856
VII-b	1	72A2_1	*Psoroma hypnorum*	OP730855
XV	2	110A, 111A	*Lecidea* sp. s. l.	OP730849, OP730850
XVI	1	24C	*Buellia* sp. s. l.	OP730848
XVII	4	94A1, 103A, 107A, 108A	*Austrolecia* sp.	OP730851, OP730852, OP730853, OP730854

## Data Availability

The sequence data presented in this study are openly available in GenBank at NCBI, accession numbers OP730747–OP830861.

## Supplementary Materials

The following supporting information can be downloaded at: https://www.mdpi.com/article/10.3390/jof9050552/s1, Table S1: List of BLAST associations, similarity percentages and top hits accession numbers for all samples analyzed in this study.
