# Supplementary material for: Survey of Lichenized Fungi DNA Barcodes on King George Island (Antarctica): An Aid to Species Discovery
Source: J Fungi (Basel). 2023 May 11;9(5):552. doi: 10.3390/jof9050552 (PMC10219471; doi:10.3390/jof9050552)
Supplement: Supplementary file 1 [file jof-09-00552-s001.zip › Table S1.pdf]

Sample group	Sample code	LCA	Determination	Top hits	Accession	Coverage range	Identity range
I	1A	Usnea	Usnea aurantiacoatra	Usnea aurantiacoatra (21)	KX147249.1, DQ534488.2, EF179799.1, …	94% - 100%	99.17% - 99.88%
				Usnea antarctica (8)	EF179796.1, KJ607898.1, HQ650616.1, …	90% - 100%	98.72% - 99.87%
I	6A	Usnea	Usnea aurantiacoatra	Usnea antarctica (22)	MG200277.1, KJ607898.1, HQ650616.1, …	91% - 100%	99.52% - 100%
				Usnea aurantiacoatra (40)	KX147245.1, DQ534488.2, EF179799.1, …	91% - 100%	99.52% - 100%
I	13D	Usnea	Usnea antarctica	Usnea antarctica (22)	MG200279.1, KJ607898.1, HQ650616.1, …	80% - 100%	99.27% - 100%
				Usnea aurantiacoatra (40)	KX147250.1, DQ534488.2, MG200305.1, …	80% - 100%	99.03% - 100%
I	14B	Usnea	Usnea aurantiacoatra	Usnea antarctica (22)	KJ607898.1, HQ650616.1, EF179795.1 …	80% - 100%	99.40% - 99.88%
				Usnea aurantiacoatra (40)	KX147249.1, DQ534488.2, EF179798.1, …	80% - 100%	99.25% - 100%
I	85A	Usnea	Usnea aurantiacoatra	Usnea antarctica (22)	KJ607899.1, HQ650616.1, MG200282.1, …	90% - 100%	98.70% - 99.86%
				Usnea aurantiacoatra (40)	KX147238.1, MG200297.1, DQ534488.2, …	90% - 100%	98.70% - 99.86%
I	3B	Usnea	Usnea antarctica	Usnea aurantiacoatra (21)	KX147249.1, DQ534488.2, EF179799.1, …	97% - 100%	99.30% - 100%
				Usnea antarctica (8)	KJ607898.1, EF179796.1, HQ650616.1, …	90% - 100%	98.95% - 99.88%
I	9C	Usnea	Usnea antarctica	Usnea aurantiacoatra (21)	KX147250.1, DQ534488.2, EF179797.1, …	96% - 100%	99.08% - 99.88%
				Usnea antarctica (8)	HQ650616.1, EF179795.1, KJ607898.1, …	89% - 100%	99.08% - 99.64%
I	22A	Usnea	Usnea antarctica	Usnea antarctica (22)	MG200276.1, KJ607898.1, HQ650616.1, …	90% - 100%	99.30% - 100%
				Usnea aurantiacoatra (40)	KX147250.1, DQ534488.2, EF179798.1, …	90% - 100%	99.03% - 99.72%
I	41A	Usnea	Usnea antarctica	Usnea aurantiacoatra (40)	KX147245.1, DQ534488.2, EF179798.1, …	90% - 100%	99.15% - 99.86%
				Usnea antarctica (22)	MG200277.1, KJ607898.1, HQ650616.1, …	90% - 100%	99.15% - 99.84%
I	71A1	Usnea	Usnea aurantiacoatra	Usnea aurantiacoatra (21)	KX147245.1, DQ534488.2, EF179798.1, …	99% - 100%	99.05% - 99.76%
				Usnea antarctica (8)	KJ607898.1, HQ650616.1, EF179795.1, …	92% - 100%	99.16% - 99.64%
I	4A	Usnea	Usnea aurantiacoatra	Neuropogon antarcticus (3)	AJ748100.1, AJ748099.1, AJ748101.1	100%	98.65% - 100%
				Usnea antarctica (16)	AB103541.1, DQ235517.1, KJ607898.1, …	88% - 100%	98.26% - 100%
				Usnea aurantiacoatra (101)	KX147251.1, DQ534488.2, KR053347.1, …	87% - 100%	96.11% - 100%
				Usnea acromelana (7)	JQ314768.1, DQ235515.1, JQ314821.1, …	87% - 95%	98.38% - 98.89%
				Usnea sp. (5)	EF492171.1, JX144648.1, MK010860.1, …	87% - 100%	95% - 98.23%
				Usnea trachycarpa (12)	JQ314702.1, EF116568.1, DQ235497.1, …	87% - 94%	97.23% - 98.01%
				Usnea ushuaiensis (1)	EF492146.1	87%	97.79%
				Usnea subantarctica (8)	DQ767961.1, EF179806.1, JF283509.1, …	90% - 100%	97.05% - 97.50%
				Usnea sphacelata (10)	DQ235495.1, EF179802.1, AB103542.1, …	92% - 96%	96.25% - 97.02%
				Usnea aff. Perpusilla (2)	DQ235512.1, DQ235510.1	90%	96.81% - 97.01%
				Usnea perpusilla (2)	DQ235490.1, DQ235488.1	90%	96.80% - 97.01%
				Usnea lambii (1)	EF179800.1	89%	96.96%
				Neuropogon trachycarpus (1)	AJ748103.1	100%	96.54%
				Neuropogon sphacelatus (1)	AJ748102.1	100%	96.53%
				uncultured Usnea (5)	KM369309.1, KM369407.1, KM369308.1, …	93% - 94%	95.68% - 95.88%
				Usnea aff. Igniaria (1)	DQ219307.1	100%	95.37%
				Usnea aff. Fragilescens (1)	MW241068.1	94%	95.31%
I	24A	Usnea	Usnea antarctica	Usnea antarctica (22)	MG200277.1, KJ607898.1, HQ650616.1, …	95% - 100%	99.54% - 100%
				Usnea aurantiacoatra (40)	KX147245.1, DQ534488.2, EF179798.1, …	95% - 100%	99.39% - 100%
I	15A	Usnea	Usnea antarctica	Usnea antarctica (22)	MG200277.1, KJ607898.1, HQ650616.1, …	100%	99.52% - 100%
				Usnea aurantiacoatra (40)	KX147245.1, MG200305.1, DQ534488.2, …	99% - 100%	99.52% - 100%
I	35A	Usnea	Usnea aurantiacoatra	Usnea antarctica (22)	KJ607898.1, EF179796.1, MG200277.1, …	96% - 100%	99.39% - 100%
				Usnea aurantiacoatra (40)	KX147253.1, DQ534488.2, EF179798.1, …	95% - 100%	99.39% - 100%
I	17A	Usnea	Usnea antarctica	Usnea antarctica (22)	KJ607898.1, MG200277.1, EF179796.1, …	100%	99.36% - 100%
				Usnea aurantiacoatra (40)	MG200296.1, KX147253.1, DQ534488.2, …	100%	99.36% - 100%
I	36A	Usnea	Usnea aurantiacoatra	Usnea antarctica (28)	MG200281.1, DQ235517.1, AB103541.1, …	80% - 99%	98.99% - 100%
				Usnea aurantiacoatra (103)	KX147245.1, MG200305.1, JF283507.1, …	80% - 99%	99% - 99.83%
				Neuropogon antarcticus (3)	AJ748100.1, AJ748099.1, AJ748101.1	80%	98.56% - 99.79%
				Usnea acromelana (3)	DQ235515.1, DQ235516.1, DQ235514.1	81%	98.57%
				Usnea sp. (4)	EF492171.1, EF492172.1, EF492174.1, …	84%	97.63% - 98.22%
				Usnea ushuaiensis (6)	EF492146.1, EF492168.1, EF492212.1, …	84%	96.64% - 97.83%
				Usnea trachycarpa (6)	DQ235497.1, DQ235496.1, EF179804.1, …	81% - 99%	95.37% - 97.75%
				Usnea perpusilla (6)	EF492147.1, EF492161.1, DQ235490.1, …	81% - 84%	96.84% - 97.63%
				Usnea lambii (13)	EF492189.1, EF492152.1, EF492199.1, …	84%	96.44% - 97.43%
				Usnea ciliata (1)	JF283511.1	85%	95.35%
				Usnea aff. Perpusilla (1)	DQ235512.1	81%	97.33%
				Usnea subantarctica (4)	JF283509.1, EF179806.1, DQ235506.1, …	81% - 85%	97.13% - 97.28%
				Usnea sphacelata (3)	DQ235495.1, EF179803.1, EF179802.1	81% - 99%	95.21% - 97.14%
I	25A	Usnea	Usnea antarctica	Usnea antarctica (22)	MG200276.1, KJ607898.1, HQ650616.1, …	90% - 100%	99.27% - 100%
				Usnea aurantiacoatra (40)	KX147250.1, DQ534488.2, EF179798.1, …	90% - 100%	98.99% - 99.71%
I	37A	Usnea	Usnea antarctica	Usnea aurantiacoatra (21)	KX147250.1, DQ534488.2, EF179799.1, …	96% - 100%	99.08% - 99.88%
				Usnea antarctica (8)	HQ650616.1, EF179795.1, KJ607898.1, …	89% - 100%	99.08% - 99.64%
I	49A	Usnea	Usnea antarctica	Usnea antarctica (22)	MG200277.1, KJ607898.1, HQ650616.1, …	95% - 100%	99.54% - 100%
				Usnea aurantiacoatra (40)	KX147245.1, DQ534488.2, EF179798.1, …	95% - 100%	99.39% - 100%
I	R12A	Usnea	Usnea antarctica	Usnea antarctica (22)	MG200277.1, KJ607898.1, HQ650616.1, …	90% - 100%	99.31% - 100%
				Usnea aurantiacoatra (40)	KX147245.1, DQ534488.2, EF179798.1, …	90% - 100%	99.31% - 100%
I	26B	Usnea	Usnea antarctica	Usnea antarctica (22)	MG200280.1, EF179795.1, KJ607898.1, …	90% - 100%	99.17% - 99.85%
				Usnea aurantiacoatra (40)	KX147250.1, DQ534488.2, EF179798.1, …	90% - 100%	98.89% - 99.58%
I	38A	Usnea	Usnea antarctica	Usnea antarctica (22)	MG200281.1, KJ607898.1, HQ650616.1, …	90% - 100%	99.01% - 100%
				Usnea aurantiacoatra (40)	KX147245.1, DQ534488.2, EF179798.1, …	90% - 100%	99.01% - 99.72%
I	105A	Usnea	Usnea antarctica	Usnea antarctica (22)	MG200277.1, KJ607898.1, HQ650616.1, …	90% - 100%	99.29% - 100%
				Usnea aurantiacoatra (40)	KX147245.1, DQ534488.2, EF179798.1, …	90% - 100%	99.29% - 100%
I	7A1	Usnea	Usnea antarctica	Usnea antarctica (22)	MG200276.1, HQ650616.1, EF179795.1, …	81% - 100%	99.15% - 100%
				Usnea aurantiacoatra (40)	KX147245.1, DQ534488.2, MG200305.1, …	81% - 100%	99.03% - 99.76%
I	29A	Usnea	Usnea antarctica	Usnea aurantiacoatra (21)	KX147245.1, DQ534488.2, EF179799.1, …	94% - 100%	99.20% - 99.89%
				Usnea antarctica (8)	HQ650616.1, EF179795.1, KJ607898.1, …	88% - 100%	98.75% - 99.76%
I	43A	Usnea	Usnea antarctica	Usnea aurantiacoatra (21)	KX147245.1, DQ534488.2, EF179799.1, …	95% - 100%	99.29% - 100%
				Usnea antarctica (8)	HQ650616.1, EF179795.1, KJ607898.1, …	92% - 100%	98.93% - 99.88%
I	40A	Usnea	Usnea antarctica	Usnea aurantiacoatra (21)	KX147245.1, DQ534488.2, EF179799.1, …	100%	99.16% - 99.88%
				Usnea antarctica (8)	KJ607898.1, HQ650616.1, EF179795.1, …	92% - 100%	99.28% - 99.76%
I	48A	Usnea	Usnea antarctica	Usnea antarctica (22)	MG200287.1, KJ607898.1, HQ650616.1, …	84% - 100%	99.36% - 100%
				Usnea aurantiacoatra (40)	KX147245.1, DQ534488.2, MG200305.1, …	84% - 100%	99.25% - 99.87%
I	20A	Usnea	Usnea antarctica	Usnea aurantiacoatra (21)	KX147250.1, DQ534488.2, EF179797.1, …	98% - 100%	98.94% - 99.76%
				Usnea antarctica (8)	HQ650616.1, EF179795.1, KJ607898.1, …	91% - 100%	99.06% - 99.53%
I	28A	Usnea	Usnea antarctica	Usnea aurantiacoatra (21)	KX147249.1, DQ534488.2, EF179799.1, …	97% - 100%	99.17% - 99.88%
				Usnea antarctica (8)	KJ607898.1, EF179796.1, HQ650616.1, …	91% - 100%	98.70% - 99.76%
I	16B	Usnea	Usnea aurantiacoatra	Usnea aurantiacoatra (40)	KX147243.1, MG200293.1, DQ534488.2, …	80% - 100%	99.10% - 99.88%
				Usnea antarctica (22)	KJ607898.1, EF179796.1, MG200277.1, …	80% - 100%	99.03% - 99.76%
I	31A	Usnea	Usnea antarctica	Usnea aurantiacoatra (21)	KX147249.1, DQ534488.2, EF179799.1, …	97% - 100%	99.06% - 99.76%
				Usnea antarctica (8)	KJ607898.1, EF179796.1, HQ650616.1, …	91% - 100%	98.70% - 99.65%
I	18C	Usnea	Usnea aurantiacoatra	Usnea aurantiacoatra (40)	KX147243.1, MG200293.1, DQ534488.2, …	89% - 100%	99.25% - 100%
				Usnea antarctica (22)	KJ607898.1, EF179796.1, MG200277.1, …	89% - 100%	99.19% - 99.87%
I	32A	Usnea	Usnea antarctica	Usnea aurantiacoatra (40)	MG200296.1, KX147249.1, DQ534488.2, …	82% - 99%	98.63% - 99.70%
				Usnea antarctica (22)	MG200277.1, KJ607898.1, EF179796.1, …	82% - 99%	98.38% - 99.55%
I	19A	Usnea	Usnea aurantiacoatra	Usnea antarctica (8)	EF179795.1, HQ650616.1, KJ607898.1, …	90% - 100%	98.95% - 99.76%
				Usnea aurantiacoatra (21)	KX147245.1, DQ534488.2, EF179799.1, …	97% - 100%	98.96% - 99.65%
I	52A4	Usnea	Usnea antarctica	Usnea antarctica (22)	KJ607898.1, EF179796.1, MG200277.1, …	91% - 100%	99.31% - 100%
				Usnea aurantiacoatra (40)	KX147253.1, DQ534488.2, MG200296.1, …	91% - 100%	99.25% - 100%

I	27A	Usnea	Usnea antarctica	Usnea aurantiacoatra (21)	KX147250.1, DQ534488.2, EF179799.1, …	94% - 100%	98.75% - 99.77%
				Usnea antarctica (8)	HQ650616.1, EF179795.1, KJ607898.1, …	88% - 100%	98.74% - 99.52%
I	45A	Usnea	Usnea antarctica	Usnea aurantiacoatra (21)	KX147245.1, DQ534488.2, EF179799.1, …	98% - 100%	99.18% - 99.88%
				Usnea antarctica (8)	HQ650616.1, EF179795.1, KJ607898.1, …	91% - 100%	99.06% - 99.76%
II	62A1	Ochrolechia	Ochrolechia frigida	Ochrolechia frigida (11)	KP314449.1, DQ534474.2, KP314435.1, …	88% - 100%	96.77% - 100%
				Ochrolechia tartarea (2)	DQ219304.1, OM397073.1	88% - 100%	96.92% - 99.82%
				Uncultured fungus (2)	KC965169.1, KC965437.1	93% - 100%	96.93% - 97.48%
II	61A5	Ochrolechia	Ochrolechia frigida	Ochrolechia frigida (13)	KP314449.1, DQ534474.2, KR017062.1, …	81% - 100%	95.12% - 100%
				Ochrolechia tartarea (2)	DQ219304.1, OM397073.1	87% - 100%	97.15% - 99.83%
				Uncultured fungus (2)	KC965169.1, KC965437.1	92% - 100%	97.17% - 97.65%
II	77A1	Ochrolechia	Ochrolechia frigida	Ochrolechia tartarea (2)	DQ219304.1, OM397073.1	87% - 100%	96.97% - 99.84%
				Ochrolechia frigida (12)	KP314449.1, DQ534474.2, HQ650675.1, …	80% - 100%	95.12% - 99.81%
				Uncultured fungus (2)	KC965169.1, KC965437.1	91% - 100%	96.87% - 97.48%
II	56A_Sup	Ochrolechia	Ochrolechia frigida	Ochrolechia frigida (11)	KP314449.1, DQ534474.2, HQ650675.1, …	87% - 100%	96.77% - 100%
				Ochrolechia tartarea (2)	DQ219304.1, OM397073.1	86% - 100%	96.92% - 99.82%
				Uncultured fungus (2)	KC965169.1, KC965437.1	91% - 100%	96.96% - 97.48%
II	44A	Ochrolechia	Ochrolechia frigida	Ochrolechia frigida (11)	KP954318.1, HQ650675.1, DQ534474.2, …	96% - 100%	95.90% - 99.76%
				Ochrolechia tartarea (2)	DQ219304.1, OM397073.1	95% - 100%	96.31% - 99.30%
				Uncultured fungus (2)	KC965169.1, KC965437.1	100%	97.18% - 97.42%
II	45B_Sup	Ochrolechia	Ochrolechia frigida	Ochrolechia tartarea (2)	DQ219304.1, OM397073.1	84% - 100%	96.71% - 100%
				Ochrolechia frigida (11)	DQ534474.2, KP314449.1, HQ650675.1, …	84% - 100%	96.57% - 100%
				Uncultured fungus (2)	KC965169.1, KC965437.1	89% - 100%	96.71% - 97.28%
II	68A3_Sup	Ochrolechia	Ochrolechia frigida	Ochrolechia frigida (11)	KP314449.1, DQ534474.2, HQ650675.1, …	87% - 100%	96.77% - 100%
				Ochrolechia tartarea (2)	DQ219304.1, OM397073.1	86% - 100%	96.92% - 99.82%
				Uncultured fungus (2)	KC965169.1, KC965437.1	91% - 100%	96.96% - 97.48%
II	29B	Ochrolechia	Ochrolechia frigida	Ochrolechia frigida (2)	DQ534474.2, HQ650675.1	100%	98.75% - 99.46%
				Ochrolechia tartarea (1)	DQ219304.1	100%	99.46%
				Uncultured fungus (2)	KC965169.1, KC965437.1	82% - 96%	96.67% - 97.39%
II	36C	Ochrolechia	Ochrolechia frigida	Ochrolechia frigida (11)	KP314449.1, DQ534474.2, HQ650675.1, …	85% - 100%	96.25% - 99.77%
				Ochrolechia tartarea (2)	DQ219304.1, OM397073.1	84% - 100%	96.42% - 99.59%
				Uncultured fungus (2)	KC965169.1, KC965437.1	90% - 100%	96.55% - 97.09%
II	54A3	Ochrolechia	Ochrolechia frigida	Ochrolechia frigida (10)	KR017062.1, KP314449.1, DQ534474.2, …	95% - 100%	96.63% - 98.02%
				Uncultured fungus (2)	KC965169.1, KC965437.1	100%	96.95% - 97.17%
				Ochrolechia tartarea (2)	DQ219304.1, OM397073.1	94% - 100%	96.33% - 97.17%
II	60A4	Ochrolechia	Ochrolechia frigida	Ochrolechia frigida (10)	KP314360.1, DQ534474.2, HQ650675.1, …	81% - 100%	96.22% - 99.6%
				Uncultured fungus (2)	KC965169.1, KC965437.1	85% - 98%	96.36% - 96.94%
				Ochrolechia tartarea (2)	DQ219304.1, OM397073.1	80% - 100%	96.36% - 99.02%
II	61A1	Ochrolechia	Ochrolechia frigida	Ochrolechia frigida (12)	KP314449.1, HQ650675.1, DQ534474.2, …	88% - 98%	95.13% - 99.81%
				Uncultured fungus (2)	KC965169.1, KC965437.1	98%	97.44% - 97.62%
				Ochrolechia tartarea (2)	DQ219304.1, OM397073.1	96% - 98%	97% - 99.63%
II	61A4	Ochrolechia	Ochrolechia frigida	Ochrolechia frigida (10)	KP314449.1, DQ534474.2, HQ650675.1, …	81% - 99%	96.27% - 99.41%
				Uncultured fungus (2)	KC965169.1, KC965437.1	85% - 98%	96.23% - 96.99%
				Ochrolechia tartarea (2)	DQ219304.1, OM397073.1	80% - 99%	96.41% - 98.87%
III	69A1	Placopsis	Placopsis antarctica	Placopsis antarctica (42)	MH670329.1, MH670328.1, MH670327.1, …	100%	98.62% - 100%
				Placopsis parellina (1)	AY212822.1	100%	100%
				Placopsis contortuplicata (11)	DQ534479.2, MH670336.1, AY212818.1, …	96% - 100%	97.06% - 98.82%
III	7B2	Placopsis	Placopsis antarctica	Placopsis antarctica (42)	MH670325.1, MH670324.1, MH670322.1, …	100%	98.41% - 100%
				Placopsis parellina (1)	AY212822.1	100%	99.80%
				Placopsis contortuplicata (11)	DQ534479.2, MH670336.1, AY212818.1, …	97% - 100%	97.22% - 98.61%
III	33D	Placopsis	Placopsis antarctica	Placopsis antarctica (42)	MH670329.1, MH670328.1, MH670327.1, …	100%	98.63% - 100%
				Placopsis parellina (1)	AY212822.1	100%	100%
				Placopsis contortuplicata (11)	DQ534479.2, MH670336.1, AY212818.1, …	95% - 100%	97.08% - 98.83%
III	13A	Placopsis	Placopsis antarctica	Placopsis antarctica (43)	MH670331.1, MH670329.1, MH670328.1, …	87% - 100%	98.26% - 100%
				Placopsis parellina (1)	AY212822.1	100%	100%
				Placopsis contortuplicata (11)	DQ534479.2, MH670336.1, KT601492.1, …	99%	97.51% - 99.01%
				Placopsis stellata (1)	AY212827.1	99%	95.53%
				Placopsis rhodophthalma (2)	AY212825.1, AY212824.1	99%	95.27%
III	75A1	Placopsis	Placopsis antarctica	Placopsis antarctica (42)	MH670329.1, MH670328.1, MH670327.1, …	100%	98.61% - 100%
				Placopsis parellina (1)	AY212822.1	100%	100%
				Placopsis contortuplicata (11)	DQ534479.2, MH670336.1, AY212818.1, …	97% - 100%	97.02% - 98.81%
III	96A	Placopsis	Placopsis antarctica	Placopsis antarctica (42)	MH670331.1, MH670329.1, MH670328.1, …	99%	98.20% - 99.64%
				Placopsis parellina (1)	AY212822.1	96%	99.44%
				Placopsis contortuplicata (11)	DQ534479.2, MH670336.1, AY212818.1, …	88% - 99%	96.44% - 98.38%
III	16A	Placopsis	Placopsis antarctica	Placopsis antarctica (42)	MH670329.1, MH670328.1, MH670327.1, …	100%	98.59% - 100%
				Placopsis parellina (1)	AY212822.1	100%	100.00%
				Placopsis contortuplicata (11)	DQ534479.2, MH670336.1, AY212818.1, …	98% - 100%	96.99% - 98.80%
III	97A1	Placopsis	Placopsis antarctica	Placopsis antarctica (42)	MH670331.1, MH670329.1, MH670328.1, …	98%	98.19% - 99.64%
				Placopsis parellina (1)	AY212822.1	95%	99.44%
				Placopsis contortuplicata (11)	DQ534479.2, KC414624.1, MH670336.1, …	87% - 98%	96.64% - 98.38%
III	10B2	Placopsis	Placopsis antarctica	Placopsis antarctica (42)	MH670325.1, MH670324.1, MH670322.1, …	100%	98.41% - 100%
				Placopsis parellina (1)	AY212822.1	100%	99.80%
				Placopsis contortuplicata (11)	DQ534479.2, MH670336.1, AY212818.1, …	97% - 100%	97.22% - 98.61%
III	6B	Placopsis	Placopsis antarctica	Placopsis antarctica (42)	MH670329.1, MH670328.1, MH670327.1, …	99%	98.74% - 100%
				Placopsis parellina (1)	AY212822.1	96%	100%
				Placopsis contortuplicata (11)	DQ534479.2, MH670336.1, AY212818.1, …	87% - 99%	96.84% - 98.92%
				Placopsis stellata (1)	AY212827.1	95%	95.14%
III	18D	Placopsis	Placopsis antarctica	Placopsis antarctica (43)	MH670331.1, MH670329.1, MH670328.1, …	84% - 100%	98.45% - 100%
				Placopsis parellina (1)	AY212822.1	100%	100%
				Placopsis contortuplicata (11)	DQ534479.2, MH670336.1, AY212818.1, …	97% - 100%	97.35% - 98.90%
				Placopsis stellata (1)	AY212827.1	99%	95.36%
III	78A	Placopsis	Placopsis antarctica	Placopsis antarctica (42)	MH670325.1, MH670324.1, MH670322.1, …	100%	98.41% - 100%
				Placopsis parellina (1)	AY212822.1	100%	99.80%
				Placopsis contortuplicata (11)	DQ534479.2, MH670336.1, AY212818.1, …	97% - 100%	97.22% - 98.61%
III	10F2	Placopsis	Placopsis antarctica	Placopsis antarctica (42)	MH670329.1, MH670328.1, MH670327.1, …	100%	98.59% - 100%
				Placopsis parellina (1)	AY212822.1	100%	100%
				Placopsis contortuplicata (11)	DQ534479.2, MH670336.1, AY212818.1, …	98% - 100%	96.99% - 98.80%
III	18E	Placopsis	Placopsis antarctica	Placopsis antarctica (42)	MH670329.1, MH670328.1, MH670327.1, …	100%	98.59% - 100%
				Placopsis parellina (1)	AY212822.1	100%	100%
				Placopsis contortuplicata (11)	DQ534479.2, MH670336.1, AY212818.1, …	98% - 100%	96.99% - 98.80%
III	80A2	Placopsis	Placopsis antarctica	Placopsis antarctica (42)	MH670325.1, MH670324.1, MH670322.1, …	100%	98.24% - 99.80%
				Placopsis parellina (1)	AY212822.1	100%	99.61%
				Placopsis contortuplicata (11)	DQ534479.2, MH670336.1, AY212818.1, …	96% - 100%	96.84% - 98.44%
III	6D	Placopsis	Placopsis antarctica	Placopsis antarctica (42)	MH670329.1, MH670328.1, MH670327.1, …	100%	99.41% - 100%
				Placopsis parellina (1)	AY212822.1	100%	100%
				Placopsis contortuplicata (11)	DQ534479.2, MH670336.1, AY212818.1, …	96% - 100%	97.06% - 98.83%
III	13B	Placopsis	Placopsis antarctica	Placopsis antarctica (42)	MH670325.1, MH670324.1, MH670322.1, …	100%	98.38% - 99.82%
				Placopsis parellina (1)	AY212822.1	96%	99.63%
				Placopsis contortuplicata (11)	DQ534479.2, KC414624.1, MH670336.1, …	88% - 100%	97.04% - 98.56%
III	81A4	Placopsis	Placopsis antarctica	Placopsis antarctica (43)	MH670331.1, MH670329.1, MH670328.1, …	81% - 100%	98.32% - 100%
				Placopsis parellina (1)	AY212822.1	100%	100%

				Placopsis contortuplicata (11)	DQ534479.2, MH670336.1, KT601492.1, …	100%	97.60% - 99.28%
				Placopsis stellata (1)	AY212827.1	100%	95.92%
				Placopsis rhodophthalma (2)	AY212824.1, AY212825.1	100%	95.20% - 95.44%
III	11D	Placopsis	Placopsis antarctica	Placopsis antarctica (42)	MH670329.1, MH670328.1, MH670327.1, …	99%	98.56% - 99.82%
				Placopsis parellina (1)	AY212822.1	95%	99.81%
				Placopsis contortuplicata (11)	DQ534479.2, KC414624.1, MH670336.1, …	90% - 99%	96.84% - 98.74%
III	19D1	Placopsis	Placopsis antarctica	Placopsis antarctica (42)	MH670329.1, MH670328.1, MH670327.1, …	100%	98.36% - 100%
				Placopsis parellina (1)	AY212822.1	97%	99.63%
				Placopsis contortuplicata (11)	DQ534479.2, KC414624.1, MH670336.1, …	89% - 100%	96.84% - 98.55%
III	83A	Placopsis	Placopsis antarctica	Placopsis antarctica (42)	MH670329.1, MH670328.1, MH670327.1, …	100%	98.41% - 99.80%
				Placopsis parellina (1)	AY212822.1	100%	99.80%
				Placopsis contortuplicata (11)	DQ534479.2, MH670336.1, AY212818.1, …	97% - 100%	96.83% - 98.61%
III	11A1	Placopsis	Placopsis antarctica	Placopsis antarctica (42)	MH670329.1, MH670328.1, MH670327.1, …	100%	98.61% - 100%
				Placopsis parellina (1)	AY212822.1	100%	100%
				Placopsis contortuplicata (11)	DQ534479.2, MH670336.1, AY212818.1, …	97% - 100%	97.02% - 98.81%
III	59A	Placopsis	Placopsis antarctica	Placopsis antarctica (42)	MH670329.1, MH670328.1, MH670327.1, …	100%	98.61% - 100%
				Placopsis parellina (1)	AY212822.1	100%	100.00%
				Placopsis contortuplicata (11)	DQ534479.2, MH670336.1, AY212818.1, …	97% - 100%	97.02% - 98.81%
III	34C	Placopsis	Placopsis antarctica	Placopsis antarctica (42)	MH670331.1, MH670329.1, MH670328.1, …	99% - 100%	98.56% - 100%
				Placopsis parellina (1)	AY212822.1	96%	99.81%
				Placopsis contortuplicata (11)	DQ534479.2, MH670336.1, AY212818.1, …	87% - 99%	96.64% - 98.74%
IV-a	49C_Bot	Caloplaca	Polycauliona regalis	Caloplaca regalis (1)	EU161240.1	100%	99.62%
				Gondwania regalis (3)	KC179103.1, KJ133463.1, KJ133462.1	95% - 98%	99.03% - 99.40%
				Gondwania cribosa (1)	KC179102.1	95%	96.40%
				Gondwania sp. (1)	KC179104.1	95%	96.19%
				Gondwania sejongensis (3)	KJ133467.1, KJ133466.1, KJ133465.1	98%	95.14% - 95.31%
				Caloplaca sublobulata (1)	DQ534455.2	99%	95.19%
IV-b	49E_Mid	Caloplaca	Caloplaca sp.	Gondwania sp. (1)	KC179104.1	83%	97.23%
				Caloplaca sublobulata (1)	DQ534455.2	100%	96.25%
				Gondwania sejongensis (1)	KJ133467.1	86%	96.01%
V-a	77A4	Umbilicaria	Umbilicaria decussata	Umbilicaria krascheninnikovii (1)	AY603134.1	100%	100%
				Umbilicaria decussata (16)	HM161510.1, AY603122.1, KY947809.1, …	92% - 100%	95.81% - 98.79%
				Umbilicaria polaris (3)	KY947830.1, KY947747.1, KY947759.1	97%	97.31% - 97.71%
				Umbilicaria aprina (16)	FN185930.1, JX036075.1, KP314390.1, …	95% - 100%	95% - 96.96%
				Umbilicaria virginis (19)	MZ244153.1, KY947767.1, AF297673.1, …	94% - 100%	96.11% - 96.84%
				uncultured eukaryote (1)	LC487926.1	100%	96.57%
				Umbilicaria proboscidea (12)	MH302526.1, FR799304.1, KY266839.1, …	90% - 100%	95.54% - 96.41%
				Umbilicaria polyrrhiza (5)	KY947838.1, JQ764737.1, MK812352.1, …	96% - 100%	95.09% - 95.77%
				Umbilicaria kappenii (3)	AY603130.1, AY603132.1, AY603131.1	93% - 99%	95.14% - 95.67%
				Umbilicaria antarctica (4)	AY603124.1, AY603128.1, AJ431605.1, …	96% - 99%	95.03% - 95.61%
				Umbilicaria havaasii (4)	KY947742.1, JQ764739.1, KY947858.1, …	95% - 100%	95.1% - 95.57%
				Umbilicaria sp.	MZ244150.1	96%	95.43%
				Umbilicaria altaiensis (1)	KY947786.1	97%	95.22%
				Umbilicaria pulvinaria (1)	KY947735.1	100%	95.14%
				Umbilicaria africana (1)	KY947844.1	99%	95.14%
				Umbilicaria formosana (1)	KY947733.1	99%	95.13%
				Umbilicaria maculata (1)	KY947794.1	97%	95.01%
V-a	93A	Umbilicaria	Umbilicaria decussata	Umbilicaria krascheninnikovii (1)	AY603134.1	100%	99.79%
				Umbilicaria decussata (16)	HM161510.1, AY603122.1, KY947809.1, …	93% - 100%	95.59% - 98.57%
				Umbilicaria polaris (3)	KY947830.1, KY947747.1, KY947759.1	99%	97.10% - 97.51%
				Umbilicaria aprina (15)	FN185930.1, JX036075.1, KP314390.1, …	97% - 100%	95.04% - 96.70%
				Umbilicaria virginis (19)	MZ244158.1, KY947767.1, AF297673.1, …	96% - 100%	95.88% - 96.62%
				uncultured eukaryote (1)	LC487926.1	100%	96.30%
				Umbilicaria proboscidea (12)	MH302526.1, FR799304.1, KY266839.1, …	91% - 100%	95.26% - 96.20%
				Umbilicaria polyrrhiza (2)	KY947838.1, JQ764737.1	97% - 100%	95.18% - 95.48%
				Umbilicaria kappenii (1)	AY603130.1	93%	95.36%
				Umbilicaria havaasii (2)	JQ764739.1, KY947742.1	96% - 100%	95.29% - 95.34%
				Umbilicaria antarctica (1)	AY603124.1	96%	95.31%
				Umbilicaria sp. (1)	MZ244150.1	98%	95.22%
				Umbilicaria hyperborea (1)	KY947744.1	98%	95.17%
				Umbilicaria altaiensis (1)	KY947786.1	99%	95.01%
V-b	62A4_Bot	Umbilicaria	Umbilicaria sp.	Umbilicaria aprina (17)	KY947808.1, JQ739981.1, JX036075.1, …	95% - 100%	95.56% - 100%
				Umbilicaria africana (3)	KY947844.1, HM161482.1, KY947743.1	100%	95.56% - 99.80%
				Umbilicaria krascheninnikovii (2)	JQ739994.1, KY947752.1	97% - 98%	95.12% - 97.11%
				Umbilicaria antarctica (35)	AY603124.1, KR053313.1, JQ739980.1, …	94% - 100%	95.16% - 96.59%
				Umbilicaria kappenii (7)	AY603130.1, AY603132.1, AY603131.1, …	91% - 100%	95.28% - 96.47%
				Umbilicaria proboscidea (11)	MK811704.1, KY266839.1, FR799304.1, …	89% - 100%	95.21% - 96.39%
				Umbilicaria formosana (3)	JQ739988.1, KY947733.1, KY947806.1	97% - 100%	95.33% - 96.07%
				Umbilicaria decussata (11)	KY947795.1, KP314408.1, HM161501.1, …	91% - 100%	95.15% - 96.05
				Umbilicaria rhizinata (1)	KY948011.1	100%	95.98%
				Umbilicaria virginis (11)	KY947832.1, KY947767.1, MZ244162.1, …	98% - 100%	95.06% - 95.96%
				uncultured eukaryote (1)	LC487926.1	100%	95.96%
				Umbilicaria havaasii (2)	KY947742.1, JQ764739.1	96% - 97%	95.01% - 95.68%
				Umbilicaria polaris (2)	KY947759.1, KY947830.1	96% - 99%	95.43% - 95.53%
				Umbilicaria maculata (1)	KY947793.1	93%	95.27%
				Umbilicaria polyrrhiza (2)	KY947838.1, JQ764737.1	97% - 100%	95.05% - 95.16%
				Umbilicaria altaiensis (1)	KY947786.1	99%	95.11%
V-b	52A3	Umbilicaria	Umbilicaria sp.	Umbilicaria aprina (17)	KY947808.1, JQ739981.1, JX036039.1, …	88% - 100%	95.21% - 98.90%
				Umbilicaria africana (3)	KY947844.1, HM161482.1, KY947743.1	99% - 100%	95.60% - 98.72%
				Umbilicaria krascheninnikovii (1)	JQ739994.1	96%	96.77%
				Umbilicaria antarctica (31)	JQ739980.1, AY603124.1, KR053313.1, …	87% - 100%	95.15% - 95.98%
				Umbilicaria rhizinata (1)	KY948011.1	100%	95.96%
				Umbilicaria formosana (2)	JQ739988.1, KY947733.1	96% - 100%	95.60% - 95.82%
				Umbilicaria kappenii (6)	AY603132.1, AY603130.1, AJ431599.1, …	84% - 100%	95.13% - 95.79%
				Umbilicaria proboscidea (8)	KY266839.1, FR799304.1, ON362174.1, …	89% - 99%	95.18% - 95.76%
				Umbilicaria decussata (11)	KP314408.1, KY947795.1, HM161501.1, …	83% - 100%	95% - 95.68%
				Umbilicaria virginis (2)	KY947832.1, KY947767.1	97% - 100%	95.29% - 95.60%
				uncultured eukaryote (1)	LC487926.1	99%	95.57%
				Umbilicaria polaris (1)	KY947759.1	97%	95.29%
VI-a	27B	Rhizocarpon	Rhizocarpon aff. geograp	Rhizocarpon geographicum (29)	DQ534482.1, KC740079.1, KC740059.1, …	100%	95.02% - 99.75%
				Rhizocarpon nidificum (1)	DQ534483.2	100%	99.75%
				Rhizocarpon aff. geographicum (21)	MZ244025.1, MZ244023.1, MZ244020.1, …	100%	95.02% - 99.25%
				Rhizocarpon sp. (1)	OM065444.1	100%	95.07%
				Rhizocarpon saanaense (2)	MZ188989.1, MZ188988.1	100%	95.04%
VI-a	66A1	Rhizocarpon	Rhizocarpon aff. geograp	Rhizocarpon geographicum (30)	DQ534482.1, KC740079.1, KC740059.1, …	99% - 100%	95.14% - 100%
				Rhizocarpon nidificum (1)	DQ534483.2	100%	100%
				Rhizocarpon aff. geographicum (21)	MZ244025.1, MZ244023.1, MZ244020.1, …	98%	95.47% - 99.59%
				Rhizocarpon sp. (2)	KP314309.1, OM065444.1	98% - 99%	95.71% - 95.74%
				Rhizocarpon saanaense (2)	MZ188989.1, MZ188988.1	99%	95.55%
VI-a	26E	Rhizocarpon	Rhizocarpon aff. geograp	Rhizocarpon geographicum (38)	DQ534482.1, KC740079.1, KC740059.1, …	99% - 100%	95.05% - 100%

				Rhizocarpon nidificum (1)	DQ534483.2	100%	100%
				Rhizocarpon aff. geographicum (21)	MZ244025.1, MZ244023.1, MZ244020.1, …	99% - 100%	95.08% - 99.48%
				Rhizocarpon sp. (2)	KP314309.1, OM065444.1	100%	95.14% - 95.36%
				Rhizocarpon saanaense (2)	MZ188989.1, MZ188988.1	100%	95.09%
VI-b	51A6	Rhizocarpon	Rhizocarpon sp.	Rhizocarpon effiguratum (1)	KC740024.1	99%	88.58%
				Rhizocarpon dispersum (2)	KC740033.1, KC740032.1	100%	88.24% - 88.40%
				Rhizocarpon norvegicum (2)	KY680775.1, KY680776.1	99% - 100%	86.97% - 88.33%
				Rhizocarpon alpicola (2)	KC740020.1, KC740019.1	98% - 99%	88.04% - 88.25%
				Rhizocarpon geographicum (80)	KC740173.1, KC757483.1, KC740097.1, …	95% - 100%	85.11% - 87.76%
				Rhizocarpon atroflavescens (4)	MK629881.1, MK625448.1, KY680777.1, …	97% - 100%	86.84% - 87.48%
				Rhizocarpon pusillum (1)	KC740025.1	97%	86.79%
				Rhizocarpon furax (1)	MT108257.1	100%	86.40%
				Rhizocarpon copelandii (3)	KU687456.1, KU687455.1, KU687447.1	97%	86.19%
				Rhizocarpon jemtlandicum (3)	KU687460.1, KU687446.1, KU687445.1	97%	85.90% - 86.09%
				Rhizocarpon geminatum var. citrinum	OK333016.1	100%	85.41%
VII-a	58A1_Bot	Psoroma hypnor	Psoroma hypnorum	Psoroma hypnorum (14)	GQ927297.1, MK811764.1, MZ159608.1, …	94% - 100%	96.09% - 100%
				Psoroma cf. hypnorum (1)	MN483079.1	99%	98.34%
				Uncultured fungus (1)	KC965717.1	100%	97.33%
VII-a	60A1	Psoroma hypnor	Psoroma hypnorum	Psoroma hypnorum (14)	GQ927297.1, MK811764.1, MZ159608.1, …	94% - 100%	95.88% - 99.79%
				Psoroma cf. hypnorum (1)	MN483079.1	99%	98.14%
				Uncultured fungus (1)	KC965717.1	100%	97.13%
VII-b	72A2_S	Psoroma	Psoroma hypnorum	Psoroma hypnorum (4)	MH802363.1, MZ159608.1, KY266971.1, …	87%	93.64% - 94.54%
				Psoroma cf. hypnorum (1)	MN483079.1	87%	94.17%
				uncultured fungus (3)	KC965717.1, KC965712.1, KC966165.1	86% - 99%	84.1% - 93.44%
				Psoroma cinnamomeum (2)	GQ927293.1, GQ927292.1	98% - 99%	84.52% - 84.75%
				Psoroma tenue (2)	GQ927291.1, GQ927290.1	98%	84.20%
				Lecanoromycetidae sp. TS-2019a (1)	MN483146.1	89%	83.73%
				Psoroma paleaceum (1)	GQ927305.1	95%	83.25%
VIII-a	48F1_Sup	Lecanora polytro	Lecanora polytropa	Lecanora polytropa (1)	DQ534470.2	100%	99.67%
VIII-b	49G	Lecanora	Lecanora sp. s.lat.	Lecanora cf. polytropa (169)	ON180214.1, ON180212.1, ON180059.1, …	90% - 93%	95.86% - 98.71%
				Lecanora polytropa (38)	KP314358.1, MZ243613.1, KP314458.1, …	91% - 99%	96.11% - 98.32%
				Lecanora fuscobrunnea (8)	GU170839.1, MK970661.1, JN873872.2, …	89% - 100%	97.35% - 97.87%
				Rhizoplaca sp. (1)	JX036118.1	92%	97.42%
				Lecanora intricata (3)	OL603995.1, OL604031.1, AY398703.1	88% - 100%	96.44% - 97.33%
				Lecanora chlorophaeodes (1)	AY398704.1	100%	96.70%
				Rhizoplaca aspidophora (1)	DQ534484.2	100%	97.16%
VIII-b	49B	Lecanora	Lecanora sp. s.lat.	Rhizoplaca aspidophora (1)	DQ534484.2	100%	100%
				Rhizoplaca sp. (1)	JX036118.1	100%	99.10%
				Lecanora fuscobrunnea (13)	MK970661.1, JN873872.2, GU170839.1, …	88% - 100%	98.59% - 99.10%
				Lecanora cf. polytropa (135)	ON180226.1, ON179998.1, ON180108.1, …	98%	95.41% - 98.63%
				Lecanora polytropa (39)	HQ650643.1, KP314321.1, MZ243599.1, …	97% - 100%	95% - 98.42%
				Lecanora intricata (3)	OL604031.1, OL603995.1, OL603994.1	86% - 100%	96.40% - 97.29%
				Lecanora cf. Intricata (1)	ON179989.1	98%	95.22%
				Lecanora chlorophaeodes (2)	AY398704.1, AF070029.1	97%	95.15%
IX	18F	Lecidella carpathi	Lecidella carpathica	Lecidella carpathica (2)	DQ534471.2, MT139649.1	100%	97.45% - 99.87%
				Sarcogyne sp. (1)	ON794211.1	95%	96.78%
				Lecidella stigmatea (3)	MT139650.1, MT139653.1, MZ159382.1	98% - 100%	96.13% - 96.65%
				Lecidella greenii (10)	NR_137582.2, JX036151.1, JX036150.1, …	81% - 100%	95.01% - 95.4%
				Lecidella patavina (1)	MT273115.1	100%	95.03%
				Lecidea sp. (1)	AY667581.1	100%	95.03%
IX	73A_Sup	Lecidella carpathi	Lecidella carpathica	Lecidella carpathica (2)	DQ534471.2, MT139649.1	100%	97.34% - 99.73%
				Sarcogyne sp. (1)	ON794211.1	94%	96.64%
				Lecidella stigmatea (3)	MT139650.1, MZ159382.1, MT139653.1	99% - 100%	96.01% - 96.54%
				Lecidella greenii (8)	NR_137582.2, JX036151.1, JX036150.1, …	81% - 100%	95.05% - 95.96%
X	24B	Candelariella flav	Candelariella flava	Candelariella flava (3)	MZ919298.1, MZ919299.1, MZ919301.1	98% - 100%	96.79% - 99.12%
X	24D	Candelariella flav	Candelariella flava	Candelariella flava (3)	MZ919298.1, MZ919299.1, MZ919301.1	98% - 100%	97.05% - 99.45%
XI	72A3	Steinera	Steinera intricata	Steinera intricata (10)	MH717151.1, MF893082.1, OP324617.1, …	96% - 100%	95.71% - 100%
				Steinera isidiata (4)	MF893086.1, NR_152550.1, MF893087.1, …	96%	95.71% - 95.92%
				Steinera subantarctica (1)	MF893108.1	96%	95.09%
XI	69A2_Bot	Steinera	Steinera intricata	Steinera intricata (10)	MH717151.1, MF893082.1, OP324617.1, …	87% - 98%	95.61% - 100%
				Steinera isidiata (4)	MF893086.1, NR_152550.1, MF893087.1, …	87%	95.61% - 95.82%
				Steinera subantarctica (1)	MF893108.1	87%	95%
XI	30A2	Steinera	Steinera intricata	Steinera intricata (10)	MH717151.1, MF893082.1, OP324617.1, …	92% - 100%	96.57% - 99.77%
				Steinera isidiata (4)	MF893086.1, NR_152550.1, MF893087.1, …	92%	97.02% - 97.25%
				Steinera subantarctica (1)	MF893108.1	92%	96.80%
				Steinera latispora (1)	MF893088.1	80%	95.54%
				Steinera pannarioides (1)	NR_152553.1	80%	95.50%
				Steinera sp. (1)	MK571785.1	92%	95.21%
XI	68A4	Steinera	Steinera intricata	Steinera intricata (10)	MH717160.1, MF893082.1, OP324617.1, …	99% - 100%	96.88% - 100%
				Steinera subantarctica (1)	MF893108.1	99%	97.73%
				Steinera isidiata (5)	MF893086.1, NR_152550.1, MF893087.1, …	99%	95.18% - 97.45%
				Steinera latispora (1)	MF893088.1	98%	95.76%
				Steinera pannarioides (4)	MF893107.1, MF893106.1, NR_152553.1, …	98%	95.74%
				Steinera sp. (2)	MK571784.1, MK571785.1	99%	96.32% - 96.34%
				Steinera lebouvieri (3)	NR_152551.1, MF893090.1, MF893089.1	98%	96.31%
XI	75A2	Steinera	Steinera intricata	Steinera intricata (10)	MH717153.1, MF893082.1, OP324617.1, …	99% - 100%	96.74% - 100%
				Steinera isidiata (5)	MF893086.1, NR_152550.1, MF893087.1, …	99%	95.90% - 98.09%
				Steinera subantarctica (1)	MF893108.1	99%	97.55%
				Steinera latispora (1)	MF893088.1	99%	95.90%
				Steinera pannarioides (4)	MF893107.1, MF893106.1, NR_152553.1, …	99%	95.90%
				Steinera lebouvieri (3)	NR_152551.1, MF893090.1, MF893089.1	98%	96.14% - 96.69%
				Steinera sp. (2)	MK571784.1, MK571785.1	97% - 98%	96.42% - 96.43%
XI	68A3_Bot	Steinera	Steinera intricata	Steinera intricata (10)	MH717153.1, MF893082.1, OP324617.1, …	99% - 100%	97.17% - 100%
				Steinera subantarctica (1)	MF893108.1	99%	98.30%
				Steinera isidiata (5)	MF893086.1, NR_152550.1, MF893087.1, …	99%	95.74% - 98.01%
				Steinera lebouvieri (3)	NR_152551.1, MF893090.1, MF893089.1	98%	96.87%
				Steinera sp. (2)	MK571784.1, MK571785.1	99%	96.32% - 96.34%
				Steinera pannarioides (4)	MF893107.1, MF893106.1, NR_152553.1, …	98%	96.30%
				Steinera latispora (1)	MF893088.1	98%	96.05%
XI	72A2_Bot	Steinera	Steinera intricata	Steinera intricata (10)	MH717160.1, MF893082.1, OP324617.1, …	100%	97.08% - 100%
				Steinera subantarctica (1)	MF893108.1	99%	97.95%
				Steinera isidiata (5)	MF893086.1, NR_152550.1, MF893087.1, …	100%	95.34% - 97.67%
				Steinera sp. (2)	MK571784.1, MK571785.1	100%	96.21% - 96.23%
				Steinera pannarioides (4)	MF893107.1, MF893106.1, NR_152553.1, …	100%	96.21%
				Steinera lebouvieri (3)	NR_152551.1, MF893090.1, MF893089.1	100%	96.21%
				Steinera latispora (1)	MF893088.1	100%	96.21%
XII	69A3_Bot	Lepraria	Lepraria sp.	Lepraria elobata (14)	AF517909.1, JQ070298.1, MG554669.1, …	88% - 100%	95.71% - 97.46%
				Lepraria cf. caesioalba (3)	AF517894.1, AF517905.1, AF517919.1	96%	95.77% - 97.45%
				Lepraria caesioalba (8)	AF517901.1, EU008610.1, MZ244060.1, …	90% - 100%	96.52% - 97.44%
				Lepraria granulata (1)	DQ914539.1	100%	97.35%

				Lepraria neglecta (33)	KC209139.1, KC209159.1, KC209164.1, …	96% - 100%	95.09% - 97.34%
				Lepraria sp. (6)	DQ914543.1, EU008630.1, OL625386.1, …	93% - 100%	95.11% - 96.94%
				Uncultured fungus (1)	KC965398.1	100%	96.72%
				Lepraria membranacea (1)	DQ534473.2	100%	96.52%
				Lepraria cacuminum (1)	AF517888.1	96%	96.19%
				Lepraria atlantica (4)	EF619550.1, EF619549.1, AF517887.1, …	96% - 100%	95.56% - 95.92%
				Lepraria humida (9)	EF619551.1, DQ401101.1, KC209155.1, …	87% - 100%	95.3% - 95.91%
				Lepraria borealis (1)	AF517908.1	96%	95.76%
				Lepraria jackii (3)	MG554672.1, KF682451.1, KF682450.1	93% - 100%	95.11% - 95.64%
XIII-a	14A	Stereocaulon	Stereocaulon sp. 1	Stereocaulon alpinum (158)	EU161238.1, DQ534486.2, MK812253.1, …	90% - 100%	95.46% - 100%
				Stereocaulon sp. (135)	MH415129.1, MN596951.1, MH415121.1, …	81% - 100%	95% - 100%
				Stereocaulon grande (1)	MK812157.1	96%	99.32%
				Stereocaulon saxatile (6)	DQ396927.1, DQ396928.1, DQ396926.1, …	99%	96.10% - 99.13%
				Stereocaulon tomentosum (9)	EU008634.1, DQ001301.1, KP314447.1, …	99% - 100%	96.52% - 97.60%
				Stereocaulon paschale (3)	DQ396897.1, HQ650690.1, DQ396924.1	99% - 100%	96.54% - 97.40%
				Stereocaulon intermedium (7)	DQ396938.1, KP348010.1, KF928152.1, …	99% - 100%	95.88% - 97.40%
				uncultured Stereocaulaceae (1)	FJ552738.1	100%	97.40%
				Stereocaulon sasakii (2)	DQ396958.1, DQ396957.1	99%	96.75% - 97.18%
				Stereocaulon myriocarpum (3)	DQ396954.1, DQ396932.1, DQ396931.1	99%	96.96% - 97.39%
				Stereocaulon alpestre (1)	DQ396893.1	99%	96.96%
				Stereocaulon botryosum (4)	DQ396952.1, KP314386.1, EU008632.1, …	99% - 100%	96.73% - 96.96%
				Stereocaulon condensatum (2)	ON228407.1, MT512646.1	100%	96.32% - 96.96%
				Stereocaulon subcoralloides (2)	EU008633.1, DQ396896.1	99%	96.75% - 96.95%
				Stereocaulon evolutum (2)	DQ396922.1, MZ159744.1	99% - 100%	96.54% - 96.74%
				Stereocaulon glabrum (7)	DQ396910.1, DQ396902.1, DQ396901.1, …	96% - 99%	95.22% - 96.52%
				Stereocaulon verruculigerum	DQ396968.1	83%	96.38%
				Stereocaulon taeniarum (1)	DQ396919.1	99%	96.32%
				Stereocaulon rivulorum (4)	MK812658.1, DQ396967.1, AF517928.1, …	99% - 100%	96.08% - 96.31%
				Stereocaulon farinaceum (1)	DQ396973.1	99%	96.30%
				Stereocaulon apocalypticum (1)	LC516206.1	100%	96.10%
				Stereocaulon curtatum (1)	DQ396949.1	99%	95.87%
				Stereocaulon exutum (1)	KP324744.1	99%	95.87%
				Stereocaulon glareosum (1)	DQ396974.1	99%	95.67%
				Stereocaulon nanodes (1)	DQ396970.1	99%	95.65%
				Stereocaulon pileatum (4)	OL625595.1, AF517927.1, MK812510.1, …	84% - 85%	95.2% - 95.45%
				Stereocaulon vesuvianum (1)	OL625606.1	81%	95.26%
				Stereocaulon verruciferum (1)	DQ396899.1	85%	95.17%
				Stereocaulon depreaultii (1)	DQ396947.1	99%	95.02%
XIII-a	1B	Stereocaulon	Stereocaulon sp. 1	Stereocaulon alpinum (154)	DQ396915.1, EU161238.1, MK812253.1, …	83% - 100%	95.11% - 100%
				Stereocaulon sp. (114)	MN839754.1, MN839753.1, MH415129.1, …	80% - 100%	95.04% - 100%
				Stereocaulon saxatile (6)	DQ396927.1, DQ396928.1, DQ396926.1, …	89%	96.37% - 99.09%
				Stereocaulon paschale (3)	DQ396897.1, HQ650690.1, DQ396924.1	89% - 98%	96.73% - 97.28%
				uncultured Stereocaulaceae (1)	FJ552738.1	98%	97.19%
				Stereocaulon tomentosum (9)	DQ396894.1, EU008634.1, KP314447.1, …	87% - 99%	95.45% - 97.09%
				Stereocaulon subcoralloides (2)	EU008633.1, DQ396896.1	87% - 89%	96.91% - 97.01%
				Stereocaulon botryosum (4)	EU008632.1, KP314389.1, DQ396952.1	86% - 96%	96.54% - 96.81%
				Stereocaulon sasakii (2)	DQ396958.1, DQ396957.1	89%	96.18% - 96.55%
				Stereocaulon taeniarum (1)	DQ396919.1	89%	96.55%
				Stereocaulon myriocarpum (3)	DQ396954.1, DQ396932.1, DQ396931.1	89%	96.36% - 96.54%
				Stereocaulon condensatum (2)	ON228407.1, MT512646.1	97% - 98%	96.36% - 96.49%
				Stereocaulon alpestre (1)	DQ396893.1	89%	96.36%
				Stereocaulon intermedium (7)	DQ396938.1, KP348010.1, KF928152.1, …	89% - 96%	95.77% - 96.36%
				Stereocaulon glabrum (7)	DQ396910.1, DQ396902.1, DQ396901.1, …	86% - 89%	95.26% - 96.36%
				Stereocaulon evolutum (2)	MZ159744.1, DQ396922.1	89% - 98%	96.36%
				Stereocaulon rivulorum (4)	MK812658.1, DQ396967.1, AF517928.1, …	84% - 97%	95.8% - 96.31%
				Stereocaulon farinaceum (1)	DQ396973.1	89%	96.17%
				Stereocaulon apocalypticum (1)	LC516206.1	99%	96.08%
				Stereocaulon nanodes (1)	DQ396970.1	89%	95.99%
				Stereocaulon exutum (1)	KP324744.1	92%	95.76%
				Stereocaulon glareosum (1)	DQ396974.1	86%	95.31%
				Stereocaulon symphycheilum (3)	MK812328.1, MK812189.1, MK811941.1	97%	95.15%
XIII-b	28B	Stereocaulon	Stereocaulon sp. 2	Stereocaulon glabrum (7)	DQ396901.1, DQ396902.1, DQ396911.1, …	95% - 98%	97.08% - 99.45%
				Stereocaulon sp. (66)	MH414985.1, MH415062.1, MN839743.1, …	85% - 100%	95.04% - 98.75%
				Stereocaulon subcoralloides (2)	EU008633.1, DQ396896.1	95% - 98%	96.17% - 96.44%
				Stereocaulon saxatile (5)	DQ396926.1, DQ396918.1, DQ396927.1, …	98%	95.08% - 96.36%
				Stereocaulon botryosum (3)	EU008632.1, KP314389.1, DQ396952.1	95% - 99%	95.06% - 96.05%
				Stereocaulon paschale (3)	DQ396897.1, HQ650690.1, DQ396924.1	98% - 100%	95.08% - 95.82%
				Stereocaulon evolutum (1)	DQ396922.1	98%	95.80%
				Stereocaulon intermedium (2)	DQ396934.1, DQ396938.1	98%	95.44% - 95.62%
				Stereocaulon alpinum (9)	EU161238.1, DQ534486.2, MK812253.1, …	80% - 100%	95.13% - 95.53%
				Stereocaulon myriocarpum (2)	DQ396932.1, DQ396954.1	98%	95.07% - 95.26%
				Stereocaulon nanodes (1)	DQ396970.1	96%	95.16%
				Stereocaulon tomentosum (2)	DQ001301.1, DQ396894.1	98% - 100%	95.06% - 95.34%
				uncultured Stereocaulaceae (1)	FJ552738.1	100%	95.15%
XIII-b	91A	Stereocaulon	Stereocaulon sp. 2	Stereocaulon glabrum (7)	DQ396902.1, DQ396911.1, DQ396909.1, …	100%	97.62% - 100%
				Stereocaulon sp. (80)	MH414985.1, MH415062.1, MN839750.1, …	94% - 100%	95.01% - 99.78%
				Stereocaulon paschale (3)	DQ396897.1, HQ650690.1, DQ396924.1	100%	96.11% - 96.54%
				Stereocaulon saxatile (6)	DQ396926.1, DQ396918.1, DQ396923.1, …	100%	95.90% - 96.54%
				Stereocaulon grande (1)	MK812157.1	85%	96.45%
				Stereocaulon subcoralloides (2)	EU008633.1, DQ396896.1	100%	96.33%
				Stereocaulon alpinum (128)	DQ396900.1, EU161238.1, MT076377.1, …	85% - 100%	95.01% - 96.32%
				Stereocaulon intermedium (2)	DQ396938.1, DQ396934.1	100%	96.10%
				Stereocaulon botryosum (2)	EU008632.1, KP314386.1	100%	95.01% - 95.90%
				Stereocaulon taeniarum (1)	DQ396919.1	100%	95.90%
				Stereocaulon evolutum (1)	DQ396922.1	100%	95.67%
				Stereocaulon myriocarpum (3)	DQ396932.1, DQ396954.1, DQ396931.1	100%	95.24% - 95.67%
				Stereocaulon tomentosum (5)	DQ001301.1, EU008634.1, DQ396908.1, …	100%	95.01% - 95.67%
				Stereocaulon sasakii (2)	DQ396957.1, DQ396958.1	100%	95.02% - 95.24%
				Stereocaulon apocalypticum (1)	LC516206.1	100%	95.03%
				Stereocaulon alpestre (1)	DQ396893.1	100%	95.03%
				uncultured Stereocaulaceae (1)	FJ552738.1	100%	95.01%
XIV	94A2	Lecanoromycetes	Myriospora sp.	Myriospora signyensis (4)	MF074120.1, MF074119.1, MF074121.1, …	99%	95.33% - 99.80%
				Lecanoromycetes sp. (2)	MH349730.1, MH071786.1	90% - 96%	99.37% - 99.55%
				Myriospora smaragdula (15)	EU870663.1, EU870664.1, DQ374141.1, …	98%	95.19% - 96.72%
				Myriospora scabrida (10)	MZ262724.1, MF074118.1, DQ525526.1, …	90% - 100%	95.51% - 96.44%
				Myriospora sp. (2)	MZ262716.1, OP162400.1	95% - 100%	95.96% - 96.41%
				Myriospora rhagadiza (1)	MF095738.1	100%	96.16%
				Myriospora myochroa (1)	MF095737.1	100%	95.56%
				Myriospora hassei (1)	MW715698.1	86%	95.35%
XV	110A	Lecidea / Sarcogy	Lecidea sp. s.l.	Lecidea sp. (24)	MK970676.1, EU263928.1, MZ243696.1, …	94% - 99%	85.43% - 87.33%
				Lecidea polypycnidophora (17)	MH231446.1, MT273102.1, EU257682.1, …	98% - 99%	86.34% - 87.15%

				Lecidea aff. laboriosa (5)	MK990116.1, MK990124.1, MK990115.1, …	94% - 99%	86.14% - 87.03%
				Lecidea perlatolica (2)	MK595453.1, MZ243725.1	99%	85.15% - 86.65%
				Lecidea andersonii (21)	MK620084.1, GU074446.1, MZ243679.1, …	94% - 99%	85.14% - 86.64%
				Lecidea aff. polypycnidophora (1)	MK990117.1	99%	86.56%
				Lecidea promiscens (1)	MK595687.1	99%	86.53%
				Lecidea laboriosa (7)	EU259902.1, MZ243714.1, MW443062.1, …	98% - 99%	85.54% - 86.51%
				Lecidea cf. polypycnidophora (1)	MK620081.1	97%	86.44%
				Lecidea aff. andersonii (3)	MK990111.1, MK990109.1, MK990110.1	93% - 100%	85.74% - 86.41%
				Sarcogyne sp. (1)	OP162364.1	99%	86.36%
				Lecidea cancriformis (3)	AY667582.1, MK970678.1, MK970677.1	99%	85.07% - 86.17%
				Lecidea hassei (1)	ON707103.1	98%	86.17%
				Lecidea violascens (1)	MZ922140.1	98%	85.80%
				Lecidea atrobrunnea (6)	GU074457.1, MT273109.1, MW443068.1, …	98% - 99%	84.95% - 85.31%
				Lecidea confluens (1)	EU263921.1	99%	85.18%
				Lecidea syncarpa (3)	MZ243739.1, MK990107.1, KF570277.1	98% - 99%	84.58% - 85.08%
				Lecidea leucothallina (2)	MG968258.1, MG968256.1	99%	84.86%
XV	111A	Lecidea / Sarcogy	Lecidea sp. s.l.	Lecidea sp. (24)	MK970676.1, EU263928.1, MZ243696.1, …	94% - 100%	85.43% - 87.33%
				Lecidea polypycnidophora (17)	MH231446.1, MT273102.1, EU257682.1, …	98% - 99%	86.34% - 87.15%
				Lecidea aff. laboriosa (5)	MK990116.1, MK990124.1, MK990115.1, …	94% - 99%	86.14% - 87.03%
				Lecidea perlatolica (2)	MK595453.1, MZ243725.1	99% - 100%	85.04% - 86.65%
				Lecidea andersonii (21)	MK620084.1, GU074446.1, MZ243679.1, …	94% - 99%	85.14% - 86.64%
				Lecidea aff. polypycnidophora (1)	MK990117.1	99%	86.56%
				Lecidea promiscens (1)	MK595687.1	99%	86.53%
				Lecidea laboriosa (7)	EU259902.1, MZ243714.1, MW443062.1, …	97% - 99%	85.54% - 86.51%
				Lecidea cf. polypycnidophora (1)	MK620081.1	97%	86.44%
				Lecidea aff. andersonii (3)	MK990111.1, MK990109.1, MK990110.1	92% - 100%	85.74% - 86.41%
				Sarcogyne sp. (1)	OP162364.1	99%	86.36%
				Lecidea cancriformis (3)	AY667582.1, MK970678.1, MK970677.1	99%	85.07% - 86.17%
				Lecidea hassei (1)	ON707103.1	98%	86.17%
				Lecidea violascens (1)	MZ922140.1	98%	85.80%
				Lecidea atrobrunnea (6)	GU074457.1, MT273109.1, MW443068.1, …	98% - 100%	84.95% - 85.20%
				Lecidea confluens (1)	EU263921.1	99%	85.18%
				Lecidea syncarpa (3)	MZ243739.1, MK990107.1, KF570277.1	97% - 100%	84.48% - 85.08%
				Lecidea leucothallina (2)	MG968258.1, MG968256.1	98%	84.86%
XVI	24C	Caliciaceae / Phys	Buellia sp. s.lat.	Buellia frigida (14)	AY667583.1, AF281307.1, JX036142.1, …	87% - 100%	87.38% - 88.18%
				Buellia sp. (5)	MK970688.1, JX036043.1, MN615683.1, …	94% - 100%	81.87% - 87.79%
				Amandinea petermannii (1)	AF250779.1	98%	87.55%
				Buellia russa (1)	DQ534454.2	100%	87.02%
				Buellia aethalea (2)	AF540496.1, AY143410.1	97% - 98%	86.14% - 87.01%
				Physciaceae sp. (1)	JF794067.1	99%	86.91%
				Buellia ocellata (2)	AF540502.1, OK332942.1	98% - 100%	85.11% - 86.36%
				Dimelaena oreina (14)	ON447572.1, OK569798.1, AJ421417.1, …	84% - 100%	82.18% - 85.45%
				Buellia sandstedei (3)	OL396606.1, OK332944.1, OK332945.1	90% - 100%	83.52% - 85.11%
				Rinodina sp. (1)	ON707084.1	99%	85.08%
				Amandinea punctata (7)	OL467351.1, HQ650627.1, EU681282.1, …	88% - 90%	83.19% - 84.62%
				Dimelaena sp. (12)	MZ229882.1, MZ229869.1, MZ229884.1, …	87% - 100%	82.01% - 84.57%
				Amandinea coniops (1)	KJ607904.1	89%	84.52%
				Amandinea lecideina (1)	MZ159589.1	90%	83.76%
				Buellia insignis (1)	OL467352.1	89%	83.69%
				Buellia microcarpa (2)	OL396670.1, MZ968996.1	99%	83.78%
				Buellia schaereri (2)	MK778592.1, AF250791.1	88% - 89%	83.37% - 83.73%
				Calicium sp. (1)	DQ812126.1	93%	83.57%
				Buellia polita (7)	MN615679.1, MN615678.1, MN615675.1, …	92%	83.26% - 83.47%
				Buellia subnumerosa (2)	LC153803.1, LC153802.1	89%	83.33%
				Buellia boseongensis (1)	MF398999.1	88%	83.19%
				Buellia geophila (1)	OL467354.1	89%	83.12%
				Buellia badia (4)	MG250192.1, MK812426.1, KX512900.1, …	100%	82.10% - 83.05%
				Calicium glaucellum (3)	AY450570.1, FR799141.1, DQ789082.1	93%	82.23% - 82.72%
				Buellia muriformis (1)	AF540501.1	93%	82.44%
				Calicium trabinellum (1)	AY450579.1	94%	82.21%
				Calicium pinicola (3)	MK811674.1, KX512917.1, KX512916.1	94%	81.78%
				Buellia halonia (1)	MN615680.1	95%	81.69%
				Buellia papillata (1)	AF250790.1	97%	81.57%
				Buellia spuria (1)	MN586930.1	100%	81.30%
				Buellia penichra (1)	AF540503.1	97%	80.88%
				Buellia arborea (1)	KX132975.1	100%	80.35%
XVII-a	108A	Letharia / Psilolec	Austrolecia sp.	Letharia vulpina (19)	FJ161632.1, KU745854.1, KX132927.1, …	85%	85.71% - 86.38%
				Letharia cf. columbiana (4)	MG645049.1, MG645045.1, MG645047.1, …	85%	85.91% - 86.35%
				Letharia cf. vulpina (3)	MG645041.1, MG645035.1, MG645042.1	85%	85.91% - 86.35%
				Letharia gracilis (2)	KJ565861.1, KJ565860.1	85%	85.91% - 86.35%
				Fungal sp. (4)	JN053159.1, JN053126.1, JN053165.1, …	85%	85.91% - 86.16%
				Letharia aff. columbiana (8)	KU745850.1, KU745811.1, MG645017.1, …	85%	85.91% - 86.16%
				Letharia lupina (36)	FJ161472.1, MG645026.1, KU745844.1, …	85%	85.68% - 86.16%
				Letharia sp. (1)	MW084670.1	85%	86.16%
				Letharia aff. vulpina (5)	MG645036.1, MG645034.1, MG645033.1, …	85%	86.16%
				Letharia columbiana (9)	KU745835.1, KU745837.1, KJ565865.1, …	85%	85.91% - 86.13%
				Letharia aff. columbiana 'barbata' (2)	MG645015.1, MG645014.1	85%	86.13%
				Letharia aff. columbiana 'rugosa' (1)	MG645030.1	85%	85.94%
				Psilolechia clavulifera (2)	MK811831.1, AY756495.1	85%	85.52%
				Psilolechia lucida (1)	MK811768.1	84%	85.33%
XVII-a	94A1	Letharia / Psilolec	Austrolecia sp.	Letharia vulpina (19)	FJ161632.1, KU745854.1, KX132927.1, …	85%	85.71% - 86.38%
				Letharia cf. columbiana (4)	MG645049.1, MG645045.1, MG645047.1, …	85%	85.91% - 86.35%
				Letharia cf. vulpina (3)	MG645041.1, MG645035.1, MG645042.1	85%	85.91% - 86.35%
				Letharia gracilis (2)	KJ565861.1, KJ565860.1	85%	85.91% - 86.35%
				Fungal sp. (4)	JN053159.1, JN053126.1, JN053165.1, …	85%	85.91% - 86.16%
				Letharia aff. columbiana (8)	KU745850.1, KU745811.1, MG645017.1, …	85%	85.91% - 86.16%
				Letharia lupina (36)	FJ161472.1, MG645026.1, KU745844.1, …	85%	85.68% - 86.16%
				Letharia aff. vulpina (5)	MG645036.1, MG645034.1, MG645033.1, …	85%	86.16%
				Letharia sp. (1)	MW084670.1	85%	86.16%
				Letharia columbiana (9)	KU745835.1, KU745837.1, KJ565865.1, …	85%	85.91% - 86.13%
				Letharia aff. columbiana 'barbata' (2)	MG645015.1, MG645014.1	85%	86.13%
				Letharia aff. columbiana 'rugosa' (1)	MG645030.1	85%	85.94%
				Psilolechia clavulifera (2)	MK811831.1, AY756495.1	85%	85.52%
				Psilolechia lucida (1)	MK811768.1	84%	85.33%
XVII-b	103A	Parmeliaceae / Ps	Austrolecia sp.	Letharia vulpina (19)	FJ161632.1, MK811758.1, KJ565917.1, …	83% - 97%	83.40% - 86.38%
				Letharia cf. vulpina (2)	MG645041.1, MG645035.1	83%	86.16% - 86.35%
				Letharia cf. columbiana (4)	MG645049.1, MG645045.1, MG645047.1, …	83%	85.91% - 86.35%
				Letharia gracilis (4)	KJ565861.1, MW084669.1, EU543560.1, …	83% - 97%	83.37% - 86.35%
				Fungal sp. (3)	JN053159.1, JN053126.1, JN053165.1	83%	86.13% - 86.16%
				Letharia lupina (25)	FJ161472.1, MG645026.1, KU745839.1, …	83% - 95%	83.76% - 86.16%
				Letharia aff. vulpina (5)	MG645036.1, MG645034.1, MG645033.1, …	83%	86.16%

				Letharia columbiana (9)	KU745837.1, KJ565865.1, KJ565853.1, …	83% - 97%	83.76% - 86.13%
				Letharia aff. columbiana (8)	KU745850.1, KU745811.1, MG645017.1, …	83% - 96%	83.56% - 86.13%
				Letharia aff. columbiana 'barbata' (2)	MG645015.1, MG645014.1	83%	86.13%
				Letharia aff. columbiana 'rugosa' (1)	MG645030.1	83%	85.94%
				Psilolechia clavulifera (2)	MK811831.1, AY756495.1	84%	85.52%
				Pleurosticta acetabulum (2)	HQ650613.1, AF058034.1	87%	85.01% - 85.41%
				Usnea wasmuthii (2)	JN086334.1, JN086331.1	87%	85.41%
				Usnea strigosa (2)	KC592273.1, MN038162.1	88%	85.07% - 85.35%
				Usnea sp. (2)	MT553305.1, MT553285.1	87% - 88%	85.19% - 85.32%
				Usnea flammea (1)	JN086293.1	88%	85.11%
				Usnea subcornuta (1)	JN086325.1	90%	85.06%
				Letharia sp. (1)	MW084670.1	97%	84.01%
				Psilolechia lucida (1)	MK811768.1	95%	82.64%
				Psilolechia leprosa (1)	JX171190.1	97%	82.23%
				Menegazzia kawesqarica (1)	KR995291.1	96%	82.21%
XVII-b	107A	Parmeliaceae / Ps	Austrolecia sp.	Pleurosticta acetabulum (4)	HQ650613.1, AY581087.1, MK812572.1, …	85%	89.36% - 89.87%
				Usnea strigosa (1)	MH310890.1	87%	89.82%
				Letharia cf. columbiana (6)	MG645043.1, MG645052.1, MG645044.1, …	95% - 99%	86.24% - 87.20%
				Letharia columbiana (10)	AF115762.2, KU745835.1, KJ565865.1, …	97% - 99%	86.21% - 86.89%
				Letharia vulpina (19)	FJ161632.1, KU745854.1, KX132927.1, …	99%	86.01% - 86.70%
				Letharia cf. vulpina (3)	MG645041.1, MG645035.1, MG645042.1	99%	86.21% - 86.67%
				Letharia gracilis (2)	KJ565861.1, KJ565860.1	99%	86.21% - 86.67%
				Fungal sp. (4)	JN053159.1, JN053126.1, JN053165.1, …	99%	86.21% - 86.47%
				Letharia aff. columbiana (8)	KU745850.1, KU745811.1, MG645017.1, …	99%	86.21% - 86.47%
				Letharia lupina (32)	FJ161472.1, MG645026.1, KU745844.1, …	99%	85.98% - 86.47%
				Letharia sp. (1)	MW084670.1	99%	86.47%
				Letharia aff. vulpina (5)	MG645036.1, MG645034.1, MG645033.1, …	99%	86.47%
				Letharia aff. columbiana 'barbata' (2)	MG645015.1, MG645014.1	99%	86.44%
				Letharia aff. columbiana 'rugosa'	MG645030.1	99%	86.24%
				Psilolechia clavulifera (2)	MK811831.1, AY756495.1	97%	86.21%
